# A novel mouse model demonstrates that oncogenic melanocyte stem cells engender melanoma resembling human disease

**DOI:** 10.1038/s41467-019-12733-1

**Published:** 2019-11-04

**Authors:** Qi Sun, Wendy Lee, Yasuaki Mohri, Makoto Takeo, Chae Ho Lim, Xiaowei Xu, Peggy Myung, Radhika P. Atit, M. Mark Taketo, Rana S. Moubarak, Markus Schober, Iman Osman, Denise L. Gay, Dieter Saur, Emi K. Nishimura, Mayumi Ito

**Affiliations:** 1 The Ronald O. Perelman Department of Dermatology, New York University, School of Medicine, New York, NY 10016 USA; 2 Department of Cell Biology, New York University, School of Medicine, New York, NY 10016 USA; 3 Department of Stem Cell Biology, Tokyo Medical and Dental University, Bunkyo-ku, Tokyo 113-8510 Japan; 4 Department of Pathology and Laboratory Medicine, Hospital of the University of Pennsylvania, Philadelphia, PA 19104 USA; 5 Department of Dermatology, Yale Cancer Center, Yale School of Medicine, New Haven, CT 06510 USA; 6 Department of Biology, Case Western Reserve University, Cleveland, OH 44106 USA; 7 Division of Experimental Therapeutics, Graduate School of Medicine, Kyoto University, Sakyo, Kyoto 606-8501 Japan; 8 Department of Pathology, New York University, School of Medicine, New York, NY 10016 USA; 9 Inserm UMR_967, CEA/DRF/IBFJ/iRCM/LRTS, 92265 Fontenay-aux-Roses cedex, France; 10 Division of Translational Cancer Research, German Cancer Research Center (DKFZ) and German Cancer Consortium (DKTK), 69120 Heidelberg, Germany; 11 Institute of Translational Cancer Research and Department of Medicine II, School of Medicine, Klinikum rechts der Isar, Technische Universität München, 81675 München, Germany

**Keywords:** Cancer models, Melanoma, Tumour heterogeneity, Skin stem cells

## Abstract

Melanoma, the deadliest skin cancer, remains largely incurable at advanced stages. Currently, there is a lack of animal models that resemble human melanoma initiation and progression. Recent studies using a *Tyr-CreER* driven mouse model have drawn contradictory conclusions about the potential of melanocyte stem cells (McSCs) to form melanoma. Here, we employ a *c-Kit-CreER*-driven model that specifically targets McSCs to show that oncogenic McSCs are a bona fide source of melanoma that expand in the niche, and then establish epidermal melanomas that invade into the underlying dermis. Further, normal Wnt and Endothelin niche signals during hair anagen onset are hijacked to promote McSC malignant transformation during melanoma induction. Finally, molecular profiling reveals strong resemblance of murine McSC-derived melanoma to human melanoma in heterogeneity and gene signatures. These findings provide experimental validation of the human melanoma progression model and key insights into the transformation and heterogeneity of McSC-derived melanoma.

## Abstract (web summary)

Currently, few mouse models exist to recapitulate human melanomagenesis. Here, the authors establish a c-Kit-CreER-driven model to target melanocyte stem cells (McSCs) and show that oncogenic McSCs give rise to epidermal melanoma that invade into the dermis, similar to human melanoma.

## Introduction

Melanoma is the deadliest form of skin cancer. The current model of human melanoma progression proposes that melanomas first arise in skin epidermis during the radial growth phase, and then invade into the dermis for continued expansion during the vertical growth phase^1^. In this latter phase, melanoma cells can establish a heterogeneous tumor composed of subpopulations with distinct proliferative, biochemical and metastatic signatures^2^. Prognosis of melanoma becomes significantly worse once the vertical growth phase is initiated, and subsequent tumor heterogeneity makes the design of effective therapeutic approaches, particularly targeted therapies, increasingly difficult^3,4^. Therefore, it is important to understand the earliest events in melanoma initiation and progression to develop strategies for early detection and intervention. Mice serve as the primary pre-clinical models in cancer research. However, to date, the validity of mouse models in studying melanoma has been questioned due to the fact that mouse melanoma arises in the dermis, while human melanoma arises specifically in the epidermis.

The capacity of melanocyte stem cells (McSCs) to create melanomas remains controversial^5,6^. To date, the study of Moon and co-workers has demonstrated the potential of hair follicle McSCs to form tumors in the well-known *Tyr-CreER; Braf*^*CA/+*^ *(Braf* ^*V600E/+*^*); Pten* ^*fl/fl*^ (Tyr-CreER:Braf:Pten) murine melanoma model^5,7^, whereas the study by Kohler et al.^6^, using the same mouse, demonstrated their lack of tumor-forming capacity. Because *Tyr-CreER* can target both McSCs located in the hair follicle and melanocytes (Mcs) in the dermis^8,9^ and melanoma forms primarily in the dermis of these mice^7^, it has proven difficult to conclusively establish the origin of melanoma using this model. Another melanoma mouse model, constitutively expressing hepatocyte growth factor/scatter factor (HGF/SF) for the migration of melanocytes to the epidermis, develops melanoma at the dermo-epidermal junction upon ultraviolet (UV) irradiation^10–13^. Although this model is thought to share more histopathologic features with human melanoma, it also cannot distinguish between epidermal and dermal melanocytes as a source for melanoma formation. Investigation for a putative vertical growth phase from epidermal melanoma in mouse melanoma studies has also been stymied using these models.

A major difficulty in the treatment of melanoma derives from the multiple levels of heterogeneity of this disease^14^. Complex phenotypic heterogeneity even within a single melanoma is common, in part because melanoma cells can dynamically and reversibly switch between differentiated and undifferentiated states, exhibiting distinct proliferative, invasive and tumor-initiating characteristics^15–18^. Without a precise understanding of the cell of origin, it remains impossible to delineate how a defined population of normal cells can initiate a transformation process that ultimately gives rise to a heterogeneous tumor. It has long been proposed that cancer cells can recapitulate embryogenesis, thus differentiated cells may acquire the multipotency of their embryonic ancestors to create heterogeneous tumors^19^. Without understanding a cellular origin of a particular melanoma, it remains impossible to test if and how this occurs after normal melanocytes acquire oncogenic mutations.

While oncogene activation and tumor-suppressor gene inactivation are thought to be the main driving events for the transformation of normal somatic cells into malignant tumor cells, the microenvironment has also been considered an active player in tumor initiation and niche signals have been shown to influence transformation in other types of cancer. For example, Wnt signal activation, driven by paracrine ligands, are required for maintenance and renewal of intestinal stem cells, but also promote their transformation during tumorigenesis^20,21^. Notch signaling, required for the proper renewal and differentiation of intestinal epithelium, is also a requisite for intestinal cancer initiation^22–24^. However, potential regenerative niche signals that synergize with oncogenic mutations to promote the transformation of normal melanocytes into melanoma remain unknown.

In this study, we generate a *c-Kit* promoter-driven model for melanoma induction^25^. We show *c-Kit* expression defines McSCs in the hair follicle (HF) and *c-Kit*^+^ follicular McSCs give rise to epidermal melanoma upon combined oncogenic Braf^V600E^ induction and Pten loss with response to normal niche signals for anagen onset. Using this model, we also clearly demonstrate that epidermal McSC-derived oncogenic Mcs invade the underlying dermis in a vertical growth phase to establish a heterogeneous melanoma remarkably similar to human melanoma.

## Results

### The *c-Kit* promoter defines follicular McSCs

**Fig. 1:**
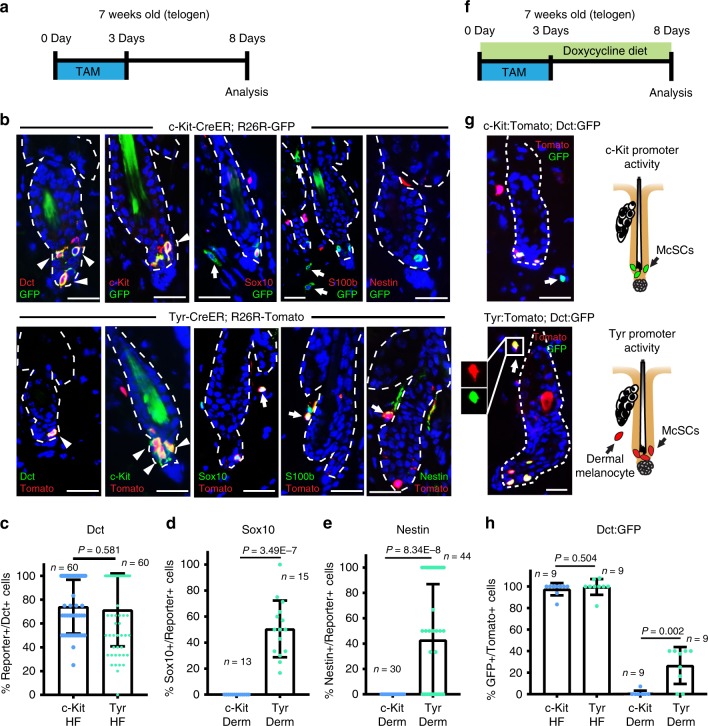
*C-Kit-CreER* targets McSCs, while *Tyr-CreER* also targets dermal melanocytic cells. **a** Schematic shows tamoxifen (TAM) treatment and analysis regimen for **b**–**e**. **b** Immunofluorescence for GFP (green) and Dct, c-Kit, Sox10, S100b, Nestin (red) in *c-Kit-CreER; R26R-GFP* skin (Top panel) and Tomato (red) and Dct, c-Kit, Sox10, S100b, Nestin (green) in *Tyr-CreER; R26R-Tomato* skin (Bottom panel). **c**–**e** Dot plot shows percentage of reporter^+^ cells in Dct^+^ cells inside the HF **c**, Sox10^+^ cells in reporter^+^ cells in the dermal compartment **d** and Nestin^+^ cells in reporter^+^ cells in the dermal compartment **e** (mean ± s.d.; 3 mice were analyzed in each group, *n* = number of hair follicles **c** or dermal areas **d**, **e** analyzed). **f** Schematic shows TAM and doxycycline(Dox) treatment and analysis regimen for **g**, **h**. **g** Immunofluorescence for GFP (green) and Tomato (red) in *c-Kit-CreER; R26R-Tomato; Dct-rtTA; tetO-H2B-GFP* skin (Top) and *Tyr-CreER; R26R-Tomato; Dct-rtTA; tetO-H2B-GFP* skin (Bottom); Schematic model showing the distribution of *c-Kit* and *Tyr* promoter active cells in right panels. **h** Dot plot shows percentage of GFP^+^ cells in Tomato^+^ cells in the HF and dermal compartment of **g** (mean ± s. d.; 3 mice were analyzed in each group, *n* = number of skin areas analyzed). Dashed line outlines the boundary of epithelium and dermis. Scale bar, 25 μm. Arrowheads point to follicular McSCs; Arrows point to GFP^+^ or Tomato^+^ cells in the dermis. *HF* hair follicle, *McSCs* melanocyte stem cells, *Derm* dermal compartment, *TAM* tamoxifen. Source data are provided as a Source Data file

To test the ability of the *c-Kit* promoter to target McSCs from the hair follicles away from the dermal melanocytes in the skin, we generated *c-Kit-CreER; R26R-mTmG* (c-Kit-CreER: R26R-GFP) mice in which membrane-bound GFP is expressed by *c-Kit*^+^ cells and their progeny following tamoxifen (TAM) treatment^26^. 7-week old mice were injected with TAM for 3 days, during a period when hair follicles in the back skin are in telogen phase and only harbor McSCs^27,28^ (Fig. 1a). McSCs are defined as dopachrome tautomerase (Dct)^+^ cells in the bulge/secondary hair germ (sHG) of the telogen hair follicle^27,28^. C-Kit and Sox10 have also been reported as McSC markers^29–31^. Immunohistochemistry revealed GFP expression in about 70% Dct^+^ McSCs in the bulge/sHG niche of the HF (Fig. 1b, c). These labeled cells persisted for >1 year ( >500 days) and were able to give rise to mature melanocytes (Supplementary Fig. 1a–c), verifying the ability of the *c-Kit* promoter to target long-lived McSCs. Immunohistochemistry revealed that GFP^+^ cells in the HF also expressed c-Kit and Sox10 (Fig. 1b). Although GFP expression was also occasionally detected in the dermis, none of the GFP^+^ dermal cells expressed melanocyte and/or melanoma markers, including Sox10, S100b, and Nestin (Fig. 1b, d, e)^32–34^. Rarely, GFP^+^CD45^+^ cells were observed in the interfollicular epidermis and dermis, consistent with the known expression of *c-Kit* in cells of hematopoietic lineage, however, the work of others has shown that this *c-Kit-CreER* line is not suitable for targeting hematopoietic stem cells (HSCs) because of low expression (Supplementary Fig. 1d, e)^35,36^. GFP expression was also occasionally detected in Keratin14 + keratinocytes in the interfollicular epidermis (Supplementary Fig. 1e). None of the GFP^+^ epidermal cells expressed Dct, consistent with the previous observations that epidermal melanocytes do not reside in the back skin of mice^28^. To further confirm that *c-Kit-CreER* does not target dermal melanocytes, we crossed *c-Kit-CreER; R26R-Tomato* reporter mice to *Dct-rtTA; tetO-H2B-GFP* mice, to GFP tag *Dct*^+^ cells upon doxycycline treatment (Fig. 1f)^37^. As expected, we observed no Tomato^+^GFP^+^ cells in the dermis of these mice (Fig. 1g, h), confirming that the *c-Kit* promoter targets only follicular McSCs.

In contrast to *c-Kit-CreER* mice, *Tyr* promoter activity was detected in both follicular McSCs and dermal melanocytes (Fig. 1b)^38^. While Dct + McSCs in the hair follicle expressed Tomato in *Tyr-CreER; R26R-Tomato* reporter mice (Fig. 1b, c), some dermal Tomato^+^ cells were also detected in these mice that expressed Sox10, S100b and Nestin (Fig. 1b, d, e; Supplementary Fig. 1f). Further, by generating *Tyr-CreER; R26R-Tomato; Dct-rtTA; tetO-H2B-GFP* mice, we detected that about 33% Tomato^+^ dermal cells were GFP^+^, indicating that the *Tyr* promoter targets dermal melanocytes (Fig. 1f–h). The combined data indicate that the *Tyr* promoter can target dermal melanocytes, while *c-Kit* promoter specifically targets follicular McSCs.

### *C-Kit*^+^ McSCs form epidermal melanomas under homeostasis

**Fig. 2:**
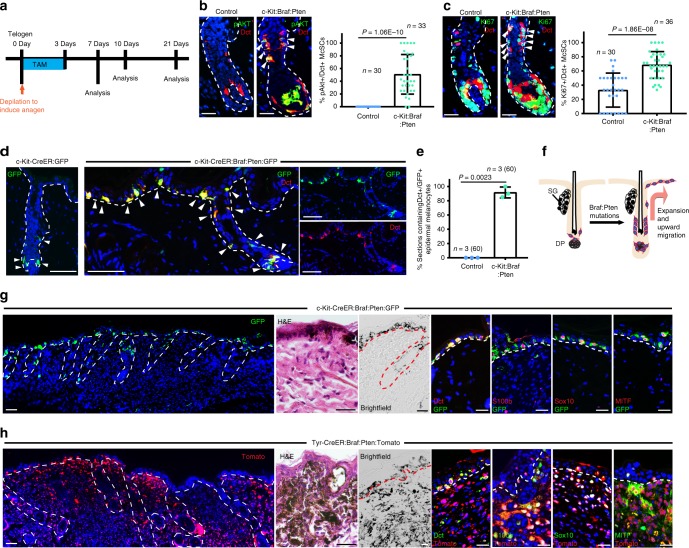
*C-Kit-CreER* targeted McSCs form epidermal melanoma following induction of Braf:Pten mutations. **a** Schematic shows TAM treatment and analysis regimen of **b**–**h**. **b** Immunofluorescence images show Dct (red) and pAKT (green) in control (left) and c-Kit-CreER:Braf:Pten (middle) skin at 7 days after initial TAM. Dot plot shows percentage of pAKT^+^ cells in Dct^+^ McSCs (right) (mean ± s.d.; *n* = 30 hair follicles from 3 control mice in control and 33 hair follicles from 3 c-Kit-CreER:Braf:Pten mice). **c** Immunofluorescence images show Dct (red) and Ki67 (green) in control (left) and c-Kit-CreER:Braf:Pten (middle) skin at 7 days after initial TAM. Dot plot shows percentage of Ki67^+^ cells in Dct^+^ McSCs (right) (mean ± s.d.; *n* = 30 hair follicles from 3 control mice in control and 36 hair follicles from 3 c-Kit-CreER:Braf:Pten mice). **d** Immunofluorescence images showing GFP (green) in *c-Kit-CreER; R26R-GFP* control mice (left panel) and GFP (green) and Dct (red) in c-Kit-CreER:Braf:Pten:GFP mice (right panels) at 10 days after initial TAM (Left). The rightmost panels show the separate channel images to demonstrate the co-localization of GFP and Dct signals. **e** Dot plot shows percentage of 6um tissue sections ( > 500  µm length) that contain Dct^+^/GFP^+^ epidermal melanocytes in control and c-Kit-CreER:Braf:Pten:GFP mice (mean ± s.d.; *n* = 3 mice, total number of tissue sections analyzed is indicated in parenthesis in each group). **f** Schematic model showing that upon oncogenic mutations, McSCs expand in the niche and migrate upward to the epidermis. **g** Immunofluorescence images showing GFP (green) and Dct, S100b, Sox10, MITF (red), H&E or brightfield images in c-Kit-CreER:Braf:Pten:GFP mice at 21 days after initial TAM. **h** Immunofluorescence images showing Tomato (red) and Dct, S100b, Sox10, MITF (green), H&E or brightfield images in Tyr-CreER:Braf:Pten:Tomato mice at 21 days after initial TAM. Dashed line outlines the boundary of epithelium and dermis. Scale bars, 25 μm, except for **d** and the leftmost panel of **g** and **h**, which are 50 μm. Arrowheads point to follicular McSCs, except in **d**, where they also define epidermal melanocytes. HF hair follicle, McSCs melanocyte stem cells, SG sebaceous gland, DP dermal papilla. Source data are provided as a Source Data file

To ask if oncogenic McSCs can form melanomas, we generated *c-Kit-CreER; Braf*^*CA/+*^*; Pten*^*fl/fl*^ (c-Kit-CreER:Braf:Pten); *R26R-mTmG* (c-Kit-CreER:Braf:Pten:GFP) mice that inducibly express *Braf*^*V600E*^ and delete *Pten* only in *c-Kit*^+^ McSCs^7^. These cells and their progeny are labeled by GFP, allowing us to follow their fate. We chose to drive McSC transformation with conditional expression of *Braf*^*V600E*^ and deletion of *Pten* (Braf:Pten) to be comparable to the well-established *Tyr-CreER; Braf*^*CA/+*^*; Pten*^*fl/fl*^ melanoma model^7^. McSCs, quiescent during telogen, are activated to proliferate and differentiate at anagen onset^27,28^. To assess the effects of oncogenic mutations on McSCs in vivo, we experimentally induced anagen onset in 3 week old mice by depilation and then induced Braf^:^Pten mutations in McSCs by TAM administration (Fig. 2a). C-Kit-CreER:Braf:Pten McSCs were analyzed at various stages of the hair cycle and compared to stage-matched control McSCs. After hair follicles entered the early anagen phase, c-Kit-CreER:Braf:Pten McSCs displayed AKT activation, demonstrated by the expression of phosphorylated AKT (pAKT), while pAKT was never observed in control McSCs at any point in the hair cycle (Fig. 2b). Coinciding with AKT activation, Dct^+^ McSCs showed increased expression of the proliferation marker Ki67 as they expanded within the stem cell region of the hair follicle compared to controls (Fig. 2c; Supplementary Fig. 2a, b). Neither expression of *Braf*^*V600E*^ nor loss of *Pten* alone was sufficient to induce the ectopic proliferation of McSCs at this stage (Supplementary Fig. 2c)^7,39,40^. Remarkably, this expansion was accompanied by pigmentation (Supplementary Fig. 3a, b) and upward migration of GFP^+^Dct^+^ melanocytes into the upper follicular compartments and the epidermis (Fig. 2d–f). The follicle-derived Mcs within the interfollicular epidermis continued to express pAKT, remained proliferative, and expanded while maintaining pigmentation (Fig. 2g; Supplementary Fig. 3c). These epidermal Mcs, not observed in control mice (Fig. 2d; Supplementary Fig. 3d), also expressed the melanocyte/melanoma markers S100b, Dct, microphthalmia transcription factor (MITF) and Sox10 (Fig. 2g; Supplementary Fig. 3e). The initial expansion of these tumorigenic epidermal melanocytes was confined to the epithelial compartment only (Fig. 2g). No Sox10^+^GFP^+^ or Dct^+^GFP^+^ cells were observed in the dermis by 2 weeks after TAM induction (Supplementary Fig. 3f).

To further verify this observation, we also examined *Tyr-CreER;Braf*^*CA/+*^*;Pten*^*fl/fl*^*;R26R-Tomato* (Tyr-CreER:Braf:Pten:Tomato) mice following TAM treatment. We experimentally induced anagen onset in telogen mice and then treated the mice with TAM for 3 days (Fig. 2a). We observed pAKT expression and increased proliferation in follicular McSCs (Supplementary Fig. 3g–i) similar to c-Kit-CreER:Braf:Pten McSCs (Fig. 2b, c), with progression of epidermal oncogenic Mcs into epidermal melanoma cells that was never observed in control mice (Fig. 2h; Supplementary Fig. 3j). It is noteworthy that the emergence of epidermal melanoma cells was associated with the robust and concomitant expansion of Tomato^+^ dermal melanoma cells in the papillary dermis, as previously observed^5,7^. Both epidermal and dermal Tomato^+^ cells expressed melanocyte/melanoma markers S100b, Dct, MITF and Sox10 (Fig. 2h; Supplementary Fig. 3e). The different phenotypes of *Tyr-CreER* to *c-Kit-CreER* driven mice suggested that dermal melanocytic subpopulation targeted by *Tyr* promoter may be responsible for the formation of dermal melanoma in Tyr-CreER:Braf:Pten mice.

Nevertheless, to ensure that *Tyr*^+^ McSCs were responsible for interfollicular epidermal expansion, we injected BrdU during anagen onset to specifically label cycling McSCs but not quiescent dermal Tyr^+^ cells and then followed with TAM-induction (Supplementary Fig. 3k)^27,41^. BrdU^+^/Tomato^+^ label retaining epidermal melanocytes were observed, verifying the origin of melanoma Mcs as follicular McSCs (Supplementary Fig. 3l–n).

### The epithelial microenvironment promotes McSC transformation

**Fig. 3:**
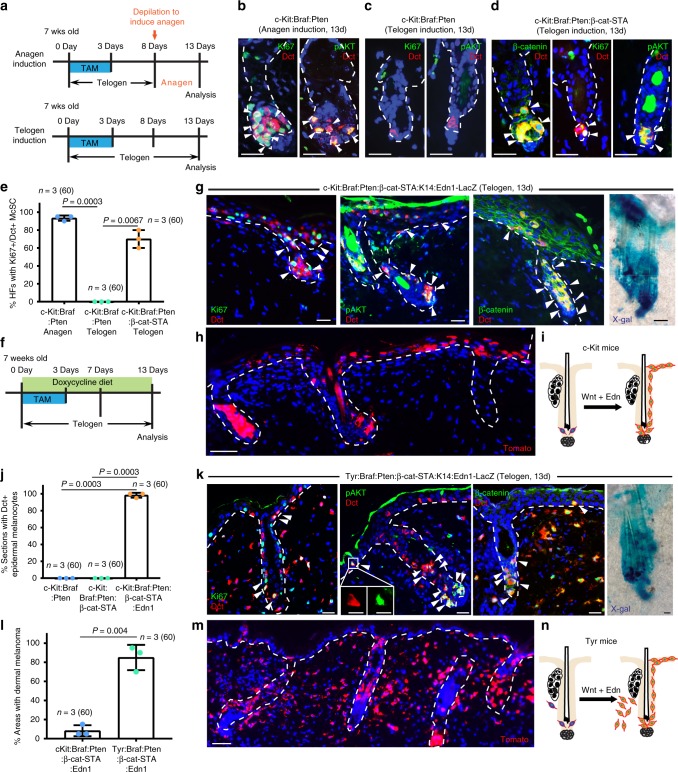
Wnt and Edn promote melanoma initiation from follicular McSCs. **a** Schematic showing TAM and depilation (top) treatment and analysis regimen for **b**–**e**. **b**–**d** Immunofluorescence for Dct (red) and Ki67, pAKT (green) in c-Kit-CreER:Braf:Pten mice with anagen induction **b**, telogen induction **c**, and c-Kit-CreER:Braf:Pten:β-cat-STA mice with telogen induction **d**. **e** Dot plot showing percentage of HFs with Ki67^+^Dct^+^ McSCs (mean ± s.d.; *n* = 3 mice, total number of hair follicles analyzed indicated in parenthesis in each group). **f** Schematic showing TAM treatment and analysis regimen for **g**–**n**. **g** Immunofluorescence for Dct (red) and Ki67, pAKT, β-catenin (green), as well as X-gal staining in c-Kit-CreER:Braf:Pten:Tomato:β-cat-STA: K14-rtTA:tetO-Edn1-lacZ mice. **h** Immunofluorescence for Tomato (red) in c-Kit-CreER:Braf:Pten:Tomato:β-cat-STA: K14-rtTA:tetO-Edn1-lacZ mice. **i** Schematic model showing that Wnt and Edn1 signals promote McSC transformation and generation of epidermal melanoma during telogen in *c-Kit* promoter driven mice. **j** Dot plot showing percentage of 6 µm tissue sections ( >500 µm length) with Dct^+^ epidermal melanocytes in mice of indicated genotypes at 13 days of telogen induction (mean ± s.d.; *n* = 3 mice, total number of tissue sections analyzed indicated in parenthesis in each group). **k** Immunofluorescence images for Dct (red) and Ki67, pAKT, β-catenin (green), as well as X-gal stained image from Tyr-CreER:Braf:Pten:Tomato:β-cat-STA: K14-rtTA:tetO-Edn1-lacZ mice. Insets in pAKT/Dct panel show separate channel images for a double positive epidermal melanocyte. **l** Dot plot showing percentage of interfollicular areas that contain > 5 Tomato^+^/Dct^+^ dermal melanoma cells (mean ± s.d.; *n* = 3 mice, total number of interfollicular areas analyzed indicated in parenthesis in each group). **m** Immunofluorescence for Tomato (red) in Tyr-CreER:Braf:Pten:Tomato:β-cat-STA: K14-rtTA:tetO-Edn1-lacZ mice. **n** Schematic model showing that Wnt and Edn1 activation in telogen induces formation of both dermal and epidermal melanomas concomitantly in Tyr promoter driven mice. Dashed line outlines the boundary of epithelium and dermis. Scale bars, 25 μm, except for **h** and **m**, which are 50 μm and 10 μm in insets of **k**. Arrowheads marker double positive follicular McSCs, except for **g** and **k**, which also point to epidermal melanocytes. Source data are provided as a Source Data file

The aberrant expansion and migration of c-Kit-CreER:Braf:Pten McSCs were typically seen during the growth phase (anagen) of the hair cycle (Fig. 3a top, b). Consistent with prior observations in the Tyr-CreER:Braf:Pten model^5,6^, Braf:Pten mutations during the telogen resting phase did not induce melanoma initiation from McSCs in either *c-Kit* or *Tyr*-driven model (Fig. 3a bottom, c; Supplementary Fig. 4a–f). Given our finding that c-Kit-CreER:Braf:Pten McSCs produce epidermal melanoma under normal conditions during anagen, we asked how mechanisms regulating normal stem cell induction might synergize with oncogenic mutations during transformation.

Because previous studies showed that paracrine expression of Wnt and endothelin (Edn) ligands by epithelial niche cells promote McSC proliferation during early anagen^42–44^, we wondered if Wnt signaling is sufficient to release Braf:Pten McSCs from their quiescent state. To test this hypothesis, we crossed *c-Kit-CreER;Braf*^*CA/+*^*;Pten*^*fl/fl*^*;R26R-Tomato* (c-Kit-CreER:Braf:Pten:Tomato) mice with mice expressing an inducible stabilized mutant form of β-catenin in which expression of stabilized β-catenin is also under control of the *c-Kit* promoter (c-Kit-CreER:Braf:Pten:Tomato:β-cat-STA mice)^45^. TAM treatment during telogen (Fig. 3a bottom) resulted in constitutive Wnt pathway activation in Braf:Pten McSCs as indicated by nuclear localization of β-catenin (Fig. 3d). These cells were shown to exit quiescence, proliferate and display AKT pathway activation, while control c-Kit:Braf:Pten McSCs remained quiescent (Fig. 3c–e; Supplementary Fig. 4f, g). *Tyr-CreER;Braf* ^*CA/+*^*;Pten*^*fl/fl*^*;β-cat-STA* (Tyr-CreER:Braf:Pten:β-cat-STA) McSCs induced during telogen phenocopied these results (Supplementary Fig. 4h). Constitutive Wnt activation in McSCs in the absence of Braf:Pten mutations had no effect, suggesting that synergy with other pathway is needed for Wnt to activate McSCs (Supplementary Fig. 4i).

Intriguingly, whereas constitutive Wnt signaling was sufficient to induce the expansion of Braf:Pten mutant McSCs during stem cell quiescence, this treatment did not promote their upward migration. However, overexpression of an additional niche factor, *Edn1*, in c-Kit-CreER:Braf:Pten:Tomato:β-cat-STA crossed to *K14-rtTA; tetO-Edn1-lacZ* mice^46,47^, promoted both expansion and upward migration of Ki67^+^pAKT^+^ McSCs in telogen (Fig. 3f–j), which is normally observed only during anagen. When we exchanged *c-Kit-CreER* for *Tyr-CreER*, epidermal melanoma formation was also observed concurrently with expansion of Tomato^+^ dermal cells (Fig. 3k–n). Of note, overexpression of epithelial *Edn1* alone during telogen did not induce proliferation, AKT pathway activation or migration to the epidermis in Braf:Pten McSCs within the quiescent follicular microenvironment (Supplementary Fig. 4j, k). These data show that Wnt and Edn signals, which are normally produced by epithelial cells during hair growth, provide the niche stimuli to promote follicular Braf:Pten McSCs to transform into melanomas. Thus, normal extrinsic signals can synergize with oncogenic mechanisms to promote McSC-derived melanoma initiation.

### Epidermal melanoma cells invade the dermis

The current model of melanoma initiation in human skin posits that melanoma arises and expands first in the epidermis (radial growth phase) before it invades into the dermis (vertical growth phase)^1^. However, the biology of dermal invasion by melanoma Mcs remains poorly understood, in part because prior mouse lineage tracing models could not distinguish between epidermal and dermal Mcs as the source of melanoma^8^. Zebrafish scale models, which can undergo a form of epidermal melanoma, also provide an imperfect likeness to the human condition because they lack strong resemblance to human skin^48^. In contrast, the c-Kit-CreER:Braf:Pten melanoma model can overcome these limitations by targeting only McSCs in the epithelium.

To determine if and how epidermal melanoma cells invade and expand in mouse dermis, we examined skin from induced c-Kit-CreER:Braf:Pten:GFP mice after establishment of epidermal melanoma. At later time points (26 days after TAM), we occasionally found foci of dermal melanoma expansion beneath the epidermis, suggestive of dermal invasion by epidermal melanoma cells (Supplementary Fig. 5a, b). Although we found very little GFP labeling in CD45^+^ cells in the skin of these mice (Supplementary Fig. 5c), most animals died at this point, likely due to defects in *c-Kit*^+^ cells in other organs, such as interstitial cells of Cajal in the gastrointestinal tract (Sun et al., unpublished).

**Fig. 4:**
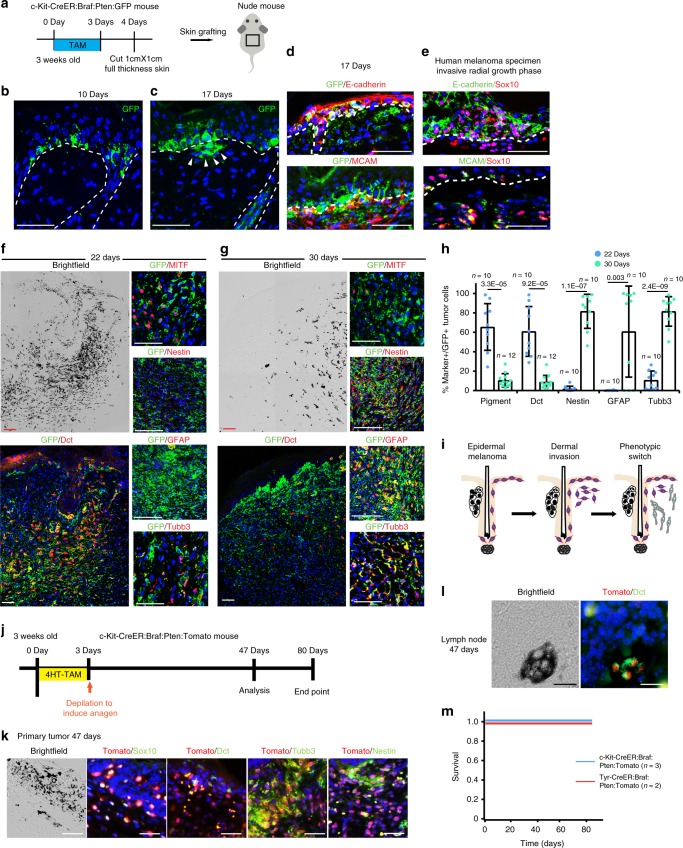
Epidermal melanoma cells invade the dermis and undergo phenotypic switch to a neuronal-like state. **a** Schematic showing TAM treatment and skin implantation onto nude mice for **b**–**h**. **b**, **c** Immunofluorescence for GFP at 10 days **b** and 17 days **c** after skin implantation in c-Kit-CreER:Braf:Pten:GFP mice. Arrowheads point to GFP^+^ melanoma cells that invade into the dermis. **d** Immunofluorescence for GFP (green) and E-cadherin, MCAM (red) at 17 days after skin implantation in c-Kit-CreER:Braf:Pten:GFP mice. **e** Immunofluorescence for Sox10 (red) and E-cadherin, MCAM (green) in human melanoma specimen during the invasive radial growth phase. **f**, **g** Immunofluorescence images for GFP (green) and Dct, MITF, Nestin, GFAP, Tubb3 (red) and brightfield images in areas of dermal melanoma at 22 days **f** and 30 days **g** after skin implantation in c-Kit-CreER:Braf:Pten:GFP mice. **h** Dot plot showing percentage of pigment, Dct, Nestin, GFAP and Tubb3 in GFP^+^ dermal melanoma cells (mean ± s.d.; 3 independent tumors from 3 mice were analyzed in each group, *n* = the number of randomly selected tumor areas analyzed. At least 1500 GFP^+^ tumor cells were analyzed in each group). **i** Schematic model illustration showing that upon oncogenic induction, McSCs first give rise to epidermal melanoma. Then melanoma cells in the epithelial compartments invade into the dermis. Finally, dermal melanoma cells undergo a phenotypic switch to acquire neuronal signatures. **j** Schematic showing 4-hydroxytamoxifen (4HT-TAM) treatment and analysis regimen. **k** Immunofluorescence images for Tomato (red) and Sox10, Dct, Tubb3, Nestin (green) and brightfield images in c-Kit-CreER:Braf:Pten:Tomato mouse skin at 47 days. **l** Immunofluorescence images for Tomato (red), Dct (green) and brightfield images in lymph node of c-Kit-CreER:Braf:Pten:Tomato mouse at 47 days. **m** Survival graph showing the days of survival of indicated mice (*n* = the number of mice). Dashed line outlines the boundary of epithelium and dermis. Scale bars, 50 μm. Source data are provided as a Source Data file

To reliably follow the phenotypic evolution of epidermal melanoma cells in mice for longer time periods, we performed full-thickness skin grafting experiments. To do this, we first treated c-Kit-CreER:Braf:Pten:GFP mice with TAM for 3 days at 3 weeks of age to genetically induce Braf:Pten mutations and GFP expression in McSCs. Skin from these animals was then grafted onto the backs of immune-deficient J/NU mice (Fig. 4a). After 10 days of engraftment, we observed a horizontal expansion of epidermal melanoma cells (Fig. 4b), similar to that seen in the c-Kit-CreER:Braf:Pten skin in situ (Fig. 2d). By 17 days, GFP^+^ melanoma cells were detected invading the graft dermis (Fig. 4c). This was observed in the interfollicular as well as follicular epithelium. Some melanoma cells, expanding within the hair follicle outer root sheath appeared to invade the underlying dermal compartment (Supplementary Fig. 6a). The GFP^+^ cells in the dermis were found to downregulate E-cadherin expression and upregulate MCAM expression (Fig. 4d), which is consistent with our and previous observations of human melanoma at the invasive radial growth phase (Fig. 4e; Supplementary Fig. 6b)^49,50^. Further investigation revealed that GFP^+^ melanoma cells that transited into the dermis maintained their pigmentation and expression of melanocytic genes, such as Dct, a melanocytic marker ubiquitously expressed in the McSC lineage in normal mice and MITF, the master transcription factor of melanocytic genes (Fig. 4f)^42,51^. These melanoma cells lacked expression of neuronal markers Nestin, GFAP and Tubb3 (Fig. 4f). By 30 days after engraftment, we found that dermal melanoma cells had significantly downregulated the expression of Dct and MITF as well as pigmentation (Fig. 4g, h). In addition, most GFP^+^ melanoma cells now expressed Nestin, GFAP and Tubb3 (Fig. 4g, h), reminiscent of human melanomas known to frequently express these neuronal markers^32,52,53^. These results indicate that melanoma cells lose their melanocytic phenotype during tumor progression and they begin to express genes including common neuronal markers (Fig. 4i).

Because nude mice lack a proper immune system, which may affect melanoma progression, we also implanted skin from c-Kit-CreER:Braf:Pten:Tomato mice onto wildtype syngeneic littermates (Supplementary Fig. 7a). These littermates formed dark melanoma by 26 days after implantation (Supplementary Fig. 7b). By 45 days, we observed Tomato^+^ tumor cells in both epidermis and dermis in the implanted area (Supplementary Fig. 7c). Similar to the results in nude mice, Tomato^+^ cells in these tumors were positive for melanoma markers such as Sox10 and heterogeneously expressing melanocytic markers Dct and pigment, as well as neuronal markers Tubb3 and Nestin (Supplementary Fig. 7c). Moreover, we detected metastasis of Tomato^+^ melanoma cells to the lymph node (Supplementary Fig. 7d). Some of these cells expressed Dct and pigment, demonstrating their melanocytic signature. No metastasis to the lung or brain was detected at this time point.

To further demonstrate that *c-Kit*^+^ McSCs can produce invasive melanoma, we topically treated c-Kit-CreER:Braf:Pten:Tomato mice with 4-hydroxytamoxifen (4HT-TAM) to induce transgene expression (Fig. 4j). By 47 days, these mice also formed heterogeneous primary melanoma tumors and lymph node metastases similar to those observed in skin transplants (Fig. 4k, l). We found very few reporter labeled CD45^+^ hematopoietic lineage cells in the dermal melanoma tumor (Supplementary Fig. 8). These topically induced mice survived at least 80 days after TAM treatment, similar to *Tyr-CreER* driven mice (Fig. 4m).

Our results suggest that this switch from a melanocytic to a neuronal-like phenotype in these mice is largely a spatially and temporally regulated process and that it coincides with the transition from the radial to the vertical growth phase.

### McSC-derived and human melanomas share molecular signatures

Our lineage tracing and phenotypic analyses suggest that the initiation and progression of murine McSC-driven melanoma resemble models of human melanoma proposed from clinical observations. To begin to understand the molecular mechanisms responsible for the transformation of McSCs to melanoma, we compared the transcriptional profiles of McSCs and their derived melanoma.

**Fig. 5:**
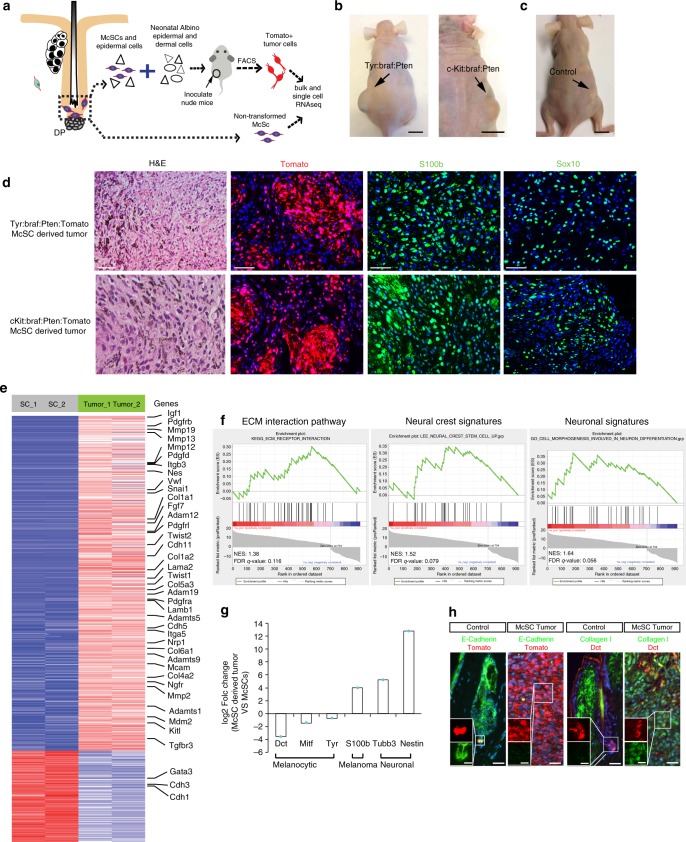
Global molecular signatures in melanoma derived from a defined population of McSCs. **a** Schematic representation of the strategy for results in Figs. 5 and 6. McSC isolation, transplantation and bulk and single cell RNA-seq comparing McSCs to their derived tumor cells. **b** Images of mice injected with McSCs from Tyr-CreER:Braf:Pten:Tomato (left) and c-Kit-CreER:Braf;Pten:Tomato (right) mice at 47 days following transplantation. Arrows point to the injection site. Scale bar: 1 cm. **c** Images of mice injected with wildtype McSCs at 47 days following transplantation. Arrows point to the injection site. Scale bar: 1 cm. **d** H&E and immunofluorescence for Tomato, S100b and Sox10 in tumors derived from McSCs of Tyr-CreER:Braf:Pten:Tomato (Top panel) and c-Kit-CreER:Braf;Pten:Tomato (Bottom panel) mice at 47 days following transplantation. Scale bar, 50 µm. **e** RNA-Seq heat map of differentially expressed genes (DEGs) shown comparison of Tyr-CreER:Braf:Pten:Tomato McSCs derived tumors to non-transformed control McSCs. Shown are DEGs with FDR < 0.02 and changes greater than 4 fold between Tyr-CreER:Braf:Pten:Tomato McSCs derived tumors and quiescent McSCs from control *Tyr-CreER*; *R26R-Tomato* mice. Red indicates high expression, while blue indicates low expression. Note that because *Tyr* and *c-Kit* promoters target the exact same population of McSCs, we chose to do RNA-seq with McSCs isolated from only one of them. **f** GSEA plots comparing the correlation of a rank-ordered list of McSC-derived tumor enriched genes to the KEGG-extracellular matrix interaction pathway (left), neural crest (middle) and neuronal gene (right) signatures. NES: normalized enrichment score. **g** Bar graph showing log_2_ fold changes of indicated markers in melanoma cells compared to normal McSCs identified in RNA-seq. **h** Immunohistochemical validation of specific RNA-seq targets in quiescent McSCs in telogen hair follicles of control mice (left) and Tyr-CreER:Braf:Pten:Tomato McSC-derived tumors (right). Inset shows separate channel images with higher magnification of boxed area. Scale bar, 25 µm; inset scale bar, 10 µm. Source data are provided as a Source Data file

To obtain tumors derived from McSCs, we isolated cells from uninduced skin epidermis including hair follicles from either *c-Kit* or *Tyr* oncogenic models and transplanted them with wildtype support cells into immunocompromised nude mice (Fig. 5a)^54^. Immediately following transplantation, we induced Braf:Pten mutations and Tomato reporter expression by TAM treatment. Transplanted McSCs from both models formed visible tumors typically by 47 days after transplantation (Fig. 5b). Consistent with a previous report, transplanted McSCs from control mice can enter the hair follicle to become both quiescent cells in the bulge and pigmented melanocytes in the bulb, but never formed tumors (Fig. 5c; Supplementary Fig. 9)^54^. Tumors from both models were indistinguishable, both containing high numbers of Tomato^+^ tumor cells expressing both S100b and Sox10 (Fig. 5d), which was expected as *Tyr* and *c-Kit* promoters targets the same population of McSCs in the hair follicle. Tomato^+^ melanoma cells derived from McSCs of Tyr-CreER:Braf:Pten:Tomato mice were then subjected to bulk and single cell RNA-sequencing (RNA-seq) (Fig. 5a; Supplementary Fig. 10a). Signatures of McSC-derived melanoma cells were compared with those of wildtype McSCs isolated from telogen HFs of *Tyr-CreER; R26R-Tomato* mice (Fig. 5a).

To identify global McSC transformation molecular signatures, we analyzed bulk RNA-seq comparisons of non-transformed McSCs and McSC-derived tumors (Supplementary Fig. 10b; Supplementary Table 1). Many of the most enriched genes in McSC-derived tumor cells were components of the extracellular matrix (ECM) including members of the integrin and collagen family and a variety of matrix-metalloproteases (MMP), indicative of epithelial–mesenchymal transition (EMT)-like changes and reminiscent of human melanoma^55,56^ (Fig. 5e, f). Gene set enrichment (GSEA) analysis also revealed a positive correlation between tumor-enriched genes and a human neural crest gene set^57^ (Fig. 5f), similar to that described in a zebrafish melanoma model^48^.

Consistent with our 30 day in vivo phenotypic analysis (Fig. 4g), neuronal markers including *Nestin*, *Tubb3*, *Gfap* and *Ngfr* were upregulated in tumors, again a noted feature of human melanomas (Fig. 5e–g). This was coupled to the downregulation of melanocytic genes (Fig. 5g). Immunohistochemical analyses with selected targets confirmed our RNA-seq results (Fig. 5h). These analyses confirmed that mouse McSC transformation involved downregulation of melanocytic genes, upregulation of ECM components, melanoma markers and neuronal/neural crest genes, all changes reminiscent of those observed in human melanoma.

### Phenotypic heterogeneity in McSC-derived melanomas

**Fig. 6:**
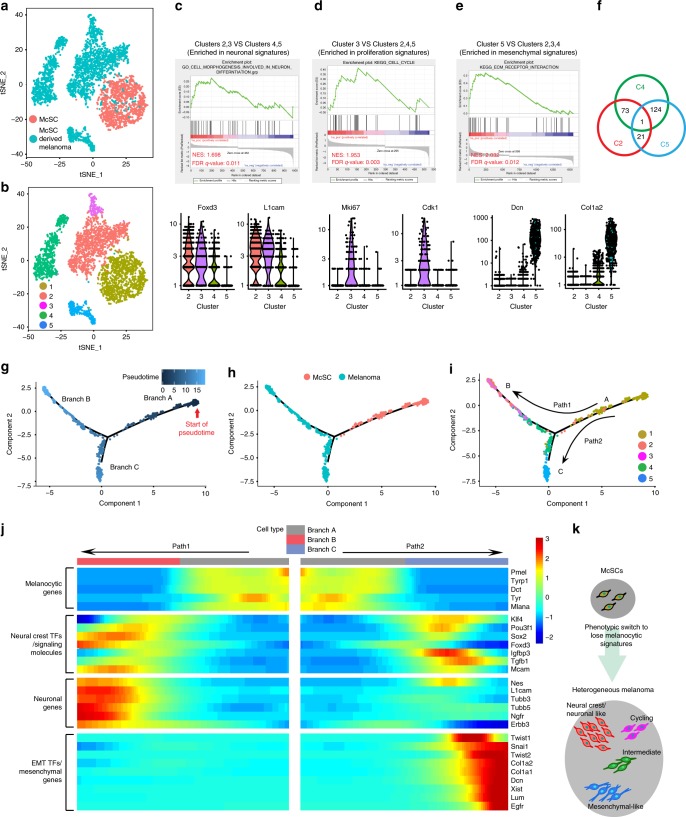
Single cell RNA-seq analyses reveal heterogeneity within a McSC-derived melanoma. **a, b** t-SNE plots showing the segregation of single cells into 5 clusters based on unbiased clustering. **a** shows the original identity of cells (McSC: red; tumor: cyan), **b** shows the 5 clusters. Because *Tyr* and *c-Kit* promoters target the exact same population of follicular McSCs, we chose to do RNA-seq with McSCs isolated from only one of them. **c** GSEA plot showing neuronal signatures enriched in cluster 2 and 3 compared to cluster 4 and 5 (Top). Violin plots show the distribution of specific neuronal genes in 4 tumor clusters (Bottom). **d** GSEA plot showing proliferation signatures enriched in cluster 3 compared to clusters 2, 4, and 5 (Top). Violin plots show the distribution of specific proliferation markers in 4 tumor clusters (Bottom). **e** GSEA plot showing mesenchymal signatures enriched in cluster 5 compared to clusters 2, 3, and 4 (Top). Violin plots show the distribution of specific mesenchymal genes in 4 tumor clusters (Bottom). **f** Diagram showing that among the top 400 enriched genes in each cluster, cluster 4 shares 73 common genes with cluster2 and 124 genes with cluster5, while cluster 2 and 5 only share 21 genes. **g**–**i** Plots showing monocle-generated pseudo-temporal trajectory. **g** is colored by pseudotime in a gradient from dark to light blue and start of pseudotime is indicated, **h** is colored by the original identify of cells, **i** is colored by the 5 clusters shown in **b**. Pseudotime ordering on McSCs and melanoma cells arranged them into 3 branches (A, B and C) and 2 paths (A to B, A to C). **j** Branched heat map showing changes of gene expression along pseudotime in both paths. The beginning of pseudotime (branch A) is in the middle of the heatmap. The path from branch A to B along pseudotime is plotted from the middle of the heatmap to the left, while the path from branch A to C is from the middle to the right. The color scale illustrates the normalized gene expression across cells. **k** Schematic model illustrating the transformation of McSCs with oncogenic mutations into heterogeneous melanoma. TFs transcription factors, EMT epithelial-mesenchymal transition, McSC melanocyte stem cell, C Cluster

To assess the heterogeneity of the McSC-initiated murine melanomas after phenotypic switch, we transplanted McSCs from Tyr-CreER:Braf:Pten:Tomato mice, induced transformation and compared gene expression of resultant melanoma cells from 3 recipients to untransformed McSCs by single cell RNA-seq (Fig. 5a). Cells segregated into 7 clusters with unsupervised clustering using Seurat analysis (Supplementary Fig. 11a–c). Two very small clusters include contaminating macrophages and keratinocytes and were excluded from the analyses (Supplementary Fig. 11d, e). Among the 5 major clusters, we found that wildtype McSCs (red) clustered into one population, suggesting that McSCs are relatively homogeneous compared with McSC-derived melanoma cells (cyan) which clustered into 4 distinctive groups (Fig. 6a, b). Little overlap was observed between normal McSC and tumor groups indicating significant transcriptional changes. Overall, all 4 melanoma clusters showed reduced expression of melanocytic markers like *Dct*, *MITF* and *Pmel*, along with elevated expression of melanoma markers (*S100b*, *MCAM* and *Sox10*) and neuronal and neural crest markers (*Nestin*, *Tubb3* and *Ngfr*) (Supplementary Fig. 12a). Our single cell RNA-seq analyses also revealed that enrichment of signatures identified by bulk analysis, including ECM-interaction pathway, neural crest signatures and neuronal differentiation signatures, were identified in all 4 tumor clusters, indicating a global shift in their expression (Table 1).

**Table 1:** Heterogeneous clusters of melanoma cells share common signatures

GSEA catogeries	Cluster2		Cluster3		Cluster4		Cluster5	
	NES	FDR q-value	NES	FDR q-value	NES	FDR q-value	NES	FDR q-value
KEGG_ECM_RECEPTOR_INTERACTION	2.410	0.000	2.280	0.000	1.930	0.001	2.430	0.000
LEE_NEURAL_CREST_STEM_CELL_UP	2.160	0.000	2.040	0.000	1.950	0.001	2.650	0.000
GO_CELL_MORPHOGENESIS_INVOLVED_IN_NEURON_DIFFERENTIATION	2.020	0.000	1.810	0.000	1.970	0.003	1.060	0.391

GSEA results of genes enriched in each cluster of melanoma cells (clusters 2–5) compared to the non-transformed McSCs (cluster1). All 4 tumor clusters (2–5) showed enrichment in the 3 key signatures observed by bulk RNA-seq (Fig. 5). NES normalized enrichment score

We also found that Clusters 2 and 3 were most enriched in a larger set of neural crest/neuronal signatures, such as *Foxd3* and *L1cam* (Fig. 6c; Supplementary Data 1). Cells in Cluster 3 also highly expressing cell cycle genes (Fig. 6d; Supplementary Data 1). By assigning cell cycle scores to each cell based on its expression of S and G2/M phase markers, we found that only Cluster 3 was enriched in cells with high S and G2/M scores (Supplementary Fig. 12b, c), suggesting that this small population of tumor cells could be responsible for tumor growth, presumably giving rise to cells in at least some of the other clusters. Cluster 5, with the lowest expression of neuronal genes, was most enriched in mesenchymal gene signatures (Fig. 6e; Supplementary Data 1). Cluster 4 appeared to be an intermediate population, sharing 124 genes with cluster5 and 73 genes with cluster2 of the top 400 enriched genes in each cluster (Fig. 6f; Supplementary Data 1). Immunohistochemical examination of 10 distinct tumors showed that in most of the tumors, Tomato^+^ tumor cells express melanocytic markers Dct and MITF, neuronal markers Nestin, Tubb3 and Gfap, mesenchymal marker Pdgfra and proliferation marker Ki67 (Supplementary Fig. 12d).

Interestingly, compared to clusters 2 and 3, clusters 4 and 5 melanoma cells were enriched in a gene set previously identified in metastatic melanoma cells from patients (Supplementary Fig. 13a)^58^. This set includes genes related to stress response, invasion and resistance to treatment with MAPK inhibitors^58^. Of note, one gene in this cluster, *SerpinE2* has recently been characterized as a marker of slow cycling cells in human melanoma that is most metastatic^59^. We found that most melanoma cells expressed *Axl*, a key marker gene for resistance to targeted therapy in human melanoma, whose expression is mostly exclusive from *Mitf*^+^ cells, consistent with previous human melanoma report (Supplementary Fig. 13b)^58,60^. Wnt and Edn pathway related genes were expressed by very few melanoma cells, suggesting that these pathways may not be required for tumor maintenance (Supplementary Fig. 13c).

To understand relatedness between clusters, we reconstructed pseudo-temporal trajectories of tumors and McSCs using Monocle. We found three main branches (A–C) (Fig. 6g–i). Branch A composed primarily of McSCs which we anticipate as the initiation site (Fig. 6g, h). Cluster4 was observed at the branching point leading to either clusters 2 & 3 with high neural crest/neuronal (Path1) gene expression or cluster5 with a strong mesenchymal (Path2) signature, suggesting that cluster4 may represent a transition state (Fig. 6i). Branched heatmap (trajectory initiation is at central location) plotted selected gene expression along pseudotime (Fig. 6j). Melanocytic genes was observed along branch A and lost in B and C. At the initiation point of branch B and C, neural crest transcription factors (TFs), such as *Sox2*, *Foxd3*, *Klf4* and *Pou3f1* and signaling molecules were upregulated. Some of these TFs and neuronal genes continue to upregulate along branch B, while in branch C, they were downregulated, followed by the upregulation of EMT transcription factors, such as *Twist1* and *Snai1* and mesenchymal genes. These data show that the transcriptional heterogeneity inherent to human melanoma, along with many of its features, is found in McSC-derived murine melanoma (Fig. 6k).

## Discussion

Despite enormous progress in our understanding of melanoma, the cellular origin of this disease remains poorly understood. Two obstacles have hampered our progress in this area: (1) the lack of specific *CreER* lines that can unequivocally mark melanocyte stem cells; and (2) a seemingly poor resemblance of mouse melanoma models to human melanoma. In using the *c-Kit-CreER* model for McSC-specific Braf:Pten activation, we were able to develop a melanoma mouse model resembling some of the key features of human melanoma. In agreement with Moon et al., we observed that tumorigenic McSCs give rise to epidermal melanomas^5^. However, Moon et al. implicated that a second hit, in this case UVB irradiation, was a requisite for formation of epidermal melanoma whereas we have demonstrated that McSCs can give rise to epidermal melanoma during normal onset of hair regeneration (Supplementary Fig. 14). Further, we have identified the niche factors involved in this process. Wnt and Edn ligands, paracrine niche factors that drive normal melanocyte differentiation during anagen^42,43^, can also fuel the transformation of McSCs into melanoma.

The microenvironment is known to play important roles in tumor initiation, progression and metastasis. Most studies have focused on how niche signals regulate behaviors of tumor cells, and the role of the microenvironment in stem cell to tumor cell transformation remains under-evaluated. Clinical observations revealed that normal donor hematopoietic cells transplanted into leukemia patients can become leukemic, indicating that an abnormal HSC niche could initiate hematopoietic malignancy^61^. This was supported by experiments in which the transplantation of normal hematopoietic cells into retinotic acid receptor gamma (Rary) deficient mice developed myeloproliferative-like disease, whereas transplantation of Rary-deficient hematopoietic cells into wildtype mice did not^62^. A recent study has further demonstrated that carcinogen-driven Bmp2 upregulation by the breast SC niche can induce the transformation of a nonmalignant mammary epithelial cell line into luminal breast tumors^63^. Although oncogenic SCs may also hijack normal niche signals, this has proven difficult to test in vivo in part due to the cellular complexity of the niche. The McSC system is unique in that McSC renewal and differentiation is driven by surrounding epithelial SCs, which can be genetically targeted for analysis^42,44,64^. Additionally, unlike most SC systems, the regenerative events of McSCs and their niche cells are synchronized and therefore easier to modulate.

A traditional view in cancer biology is that tumorigenesis recapitulates embryonic development^19^. This concept has been validated in the zebrafish model, in which Kaufman et al. showed that loss of melanocyte signatures and emergence of neural crest signatures precede the expansion of melanoma^48^. In agreement with this concept, our study showed that transformed McSCs may overcome the restrictions of their adult lineage and re-acquire the plasticity of their embryonic neural crest lineage origin. Nevertheless, our model shows that such a phenotypic change occurs after epidermal melanoma expansion and subsequent invasion into the dermis, instead of being the initial step for transformation. We speculate that the observed difference may be due to differing microenvironment and structural features in zebrafish scales and mammalian skin.

Phenotypic switching is a well-known phenomenon in human melanoma and is thought to underlie the phenotypic intra-tumor heterogeneity and resultant drug resistance of an established melanoma^4,17^. Human melanoma heterogeneity has been studied at the transcriptional level in 19 patient-derived melanoma tumors analyzed by single cell RNA-seq^58^. In this work, the intra-tumoral heterogeneity correlated with 1) segregation of proliferative and dormant cells; 2) heterogeneity of *Mitf*^+^/*Axl*^**−**^ and *Axl*^+^/*Mitf*^**−**^ populations resistant to MAPK inhibitor therapy; 3) Identification of a cluster of enriched genes related to stress response, therapy resistance and migration. In our single cell analysis of McSC-derived tumors, we observed all 3 conditions, suggesting that our SC-derived tumors phenocopy heterogeneous human melanoma populations. In the clinic, lack of response to therapies frequently correlates with tumor heterogeneity in which cell subsets respond unequally to therapy. We predict that the melanoma tumor in our model is resistant to Braf inhibitors, such as Vemurafenib/Dabrafenib given the fact that most of melanoma cells in our tumor is *Axl*^+^/*Mitf*^−58^.

The comparison of our *c-Kit-CreER* and *Tyr-CreER* driven melanoma models suggest that dermal melanocytic cells may also be a source of melanoma. The exact distribution, composition and characteristics of dermal melanocytes are not well studied. Yet, it is noteworthy that the presence of dermal stem cells with the ability to regenerate melanocytes have been identified^65,66^, and their melanoma-forming capacity has been postulated^67–69^. Future studies identifying specific tool to target dermal melanocytes/stem cells is needed to determine their ability to form melanoma. Another intriguing question is whether fully differentiated melanocytes can produce melanoma^5,6^. A previous important observation suggests that pigmented epidermal melanocytes produce dermal melanoma in the tail skin of Tyr-CreER:Braf:Pten mice. The development of definitive genetic tools to specifically trace differentiated melanocytes during tumorigenic transformation and dermal invasion may effectively validate such observations in the future.

## Methods

### Mice

All animal experiments were performed in compliance with all relevant ethical regulations for animal testing and research and in accordance with animal protocols approved by the Institutional Animal Care and Use Committee (IACUC) at New York University (NYU) School of Medicine.

*C-Kit-CreER* mice were obtained from Dr. Dieter Saur^25^. *Tyr-CreER*^8^, *Tyr-CreER;Braf* ^*CA/+;*^*Pten* ^*fl/fl*7^, *R26R-Tomato*^38^, *R26R-mTmG*^26^, *K14-rtTA*^46^, *tetO-Edn1-lacZ*^47^, and J:NU mice were purchased from The Jackson Laboratory. *Dct-rtTA; tetO-H2B-GFP* (iDCT-GFP) mice were obtained from NCI Mouse Repository^37^. *β-catenin* ^*fl(ex3)/+*^ (β-cat-STA) mice were from Dr. M. Mark Taketo^45^. Mice were bred and crossed in-house to obtain experimental and control animals in mixed backgrounds. Mice of both genders were used in all experiments, except for immunodeficient J:NU mice, which were all females.

To induce Cre recombination, Tamoxifen (TAM) (Sigma-Aldrich) treatment was performed by intraperitoneal (i.p.) injection (0.1 mg/g body weight) of a 20 mg/ml solution in corn oil per day^70^ except for one experiment (Fig. 4j–m) in which mice were topically treated with 4-hydroxytamoxifen (4HT-TAM) (Sigma-Aldrich). Mice driven by *c-Kit-CreER* were injected with TAM for 3 days, mice driven by *Tyr-CreER* were injected with TAM for 3 or 7 days (see experiment scheme for each experiment), immunodeficient nude mice transplanted with McSCs were injected TAM for 7 days. Topical administration of 4-HT was conducted by preparing a 50 mg/ml solution of 4-HT in dimethylsulfoxide (DMSO). In total 10 μl of the 4-HT solution was topically applied on a defined area of 2 × 2 cm on the depilated back skin daily for 3 days.

Mice that contain tetracycline-inducible transgenes were given doxycycline (1 g/kg; BioServ) containing diet. For BrdU administration, mice were injected daily intraperitoneally during indicated periods with 10 mg ml^−1^ BrdU (Sigma) solution in PBS at 50 μg/g body weight^54^.

To experimentally induce the synchronized hair follicle cycle, hair depilation was performed during telogen phase (3 weeks or 7 weeks old) to induce anagen phase. We applied hair removing wax (Sally Hansen) to dorsal skin and gently peeled it off or physically plucking hair by hand. Skin biopsies were obtained from back skin of mice at various time points after treatments. Mice are anesthetized by inhalation of isoflurane during both procedures.

### Skin grafting

Full-thickness skin grafting was performed as published with slight modifications^71^. Donor mice were injected TAM for 3 days at 3 weeks old. At 1 day after last TAM treatment, both donor and recipient mice were anesthetized by inhalation of isoflurane. A piece of 1.5 cm × 1.5 cm full-thickness back skin was excised with surgical scissors from donor mice and spread onto a sterile gauze soaked with PBS. A piece of 1.5 cm × 1.5 cm skin was excised from the back of recipient mice. The donor skin was then placed and sutured onto the recipient mice by 8 interrupted sutures of 6–0 silk (Ethilon). Skin biopsies were obtained from grafted skin of mice at least 10 days after grafting.

### Immunofluorescence

For paraffin sections, tissue (skin, tumor or lymph node) excised from mice was fixed overnight in 4% paraformaldehyde (PFA) at 4 °C. After sequential dehydration in increasing concentrations of ethanol and xylene, the tissue was embedded in paraffin. Paraffin sections were cut at 6 μm, deparaffinized and microwaved in 10 mM Tris and 1 mM EDTA (pH 8.0) for antigen retrieval. Tissue sections were then incubated with the primary antibodies listed below for 2 h at room temperature or overnight at 4 °C in PBT (PBS + 0.1% Triton-X100) + 10% FBS, followed by Alexa 488– or 594–conjugated secondary antibodies (1:200; Thermo Fisher #A-11055, A-11058, A-21206, A-21207, A-21202, A-21203, A-11039 or A-21208). Following primary antibodies were used: goat anti-Dct (1:100; Santa Cruz #sc-10451), rabbit anti-GFP (1:400; Abcam #ab290), chicken anti-GFP (1:400; Abcam #ab13970), rabbit anti-S100b (1:100; Dako #Z0311), goat anti-sox10 (1:00; Santa Cruz #sc-17342), mouse anti-β-catenin (1:400; Sigma #C7207), rabbit anti-Ki67 (1:100; Abcam #15580), Mouse anti-Tubb3 (1:1000; Biolegend #801201), Rabbit anti-Tomato (1:1000; Rockland #600–401–379), Mouse anti-Tomato (1:500; Thermo Fisher #MA5–15257), rabbit anti-Keratin14 (1:300; Covance #PRB-155P), mouse anti-E-cadherin (1:500; BD Biosciences #610181), rat anti-BrdU (1:100; Abcam #ab6326), rabbit anti-MCAM (1:100; Abcam #75769), rabbit anti-GFAP (1:100; Dako #Z0334). Incubation of three other primary antibodies, mouse anti-Nestin (1:100; Abcam #ab6142), mouse anti-MITF (1:100; Abcam #ab12039) and rabbit anti-pAKT XP (1:100; Cell Signaling #4060 S) were followed by incubation with biotin-conjugated secondary antibodies (1:100; Vector Laboratories #BA-2000 or BA-1100), and then streptavidin conjugated to 488– or 594– (1:200; Thermo Fisher # S32354 or S32356) was used as the final amplification step. For Tomato/BrdU double color immunofluorescence, the Tomato was stained first, and then the slides were treated with 2 N HCL for 1 h at 50 °C and then stained BrdU.

Immunofluorescence was also performed on formalin-fixed, paraffin-embedded human melanoma samples. The samples were obtained from Interdisciplinary Melanoma Cooperative Group (IMCG) biospecimen database of New York University (NYU) Langone Medical Center and evaluated by an attending pathologist as superficial-spreading melanoma. The samples that contain a few dermal invasions (invasive radial growth phase) were used for analysis.

For frozen sections, skin tissues were fixed in 4% PFA for 10 min at 4 °C and then embedded in OCT compound (Sakura) and stored immediately at −80 °C. In total 10 μm sections were made and incubated with primary antibodies listed below in PBT + 10% FBS for 2 h at room temperature. After incubation with Rat anti-c-Kit (1:100; BD Biosciences #553868) or Rat anti-Pdgfra (1:100, eBioscience #14–1401–82) primary antibody, the sections were incubated with biotin-conjugated secondary antibody (1:100; Vector Laboratories #BA-9400) for 1 h and then streptavidin conjugated to Alexa 647 (1:200; Thermo Fisher #S32357) for 30 min. After incubation with Biotin anti-mouse CD45 (1:100; Biolegend #103103) primary antibody, the sections were incubated with then streptavidin conjugated to Alexa 647 (1:200; Thermo Fisher #S32357) for 30 min.

Sections were counterstained with 4′,6-diamidine-2′-phenylindole dihydrochloride (DAPI). All immunofluorescence were analyzed and photographed with a Nikon Eclipse Ti microscope. The images were processed using NIS-Elements software and Adobe Photoshop.

### X-gal staining

Skin tissue was fixed in 4% PFA for 30 min at 4 °C and processed for β-galactosidase activity overnight at 37 °C and samples imaged using a Nikon Eclipse Ti microscope.

### McSCs isolation

Mice were sacrificed and their fur clipped. Mice were then rinsed in betadine, followed by 70% ethanol. Scalpel blades were used to remove subcutaneous fat and skin was rinsed in PBS and cut into 0.5 cm×0.5 cm pieces, followed by incubation in 0.25% Trypsin for 2 hr at 37 °C. Epidermis was separated from the dermis using forceps and scalpel blades and the epidermis was chopped finely and transferred into Media A (DMEM, 10% FBS, 1x penicillin/streptomycin). The epidermal and McSC mixture was stirred at room temperature (RT) for 20 min. The obtained McSC suspension was filtered through a 70 µm nylon filter and centrifuged at 200 × g for 7 min and resuspended in Media A.

### McSC transplantation into nude mice

Aliquot of McSC suspension were stained with Rat anti-c-Kit (1:100 in PBS + 10% FBS) for 15 minutes at room temperature and washed twice in PBS. Followed by incubation with biotinylated secondary antibodies (1:100) at RT for 15 min and then incubated with streptavidin-conjugated Alexa 594 (1:200) for 15 min at RT. Cells were counterstained with DAPI and live melanocytes were identified and quantified based on c-Kit positivity. In total 2.5 × 10^4^ melanocytes were combined with the neonatal epidermal (1 × 10^6^ cells) and dermal (1 × 10^7^ cells) cells from wildtype albino neonates that were prepared according to previous report^72^. The mixture of cells was centrifuged at 200 × *g* and pellet was re-suspended in 30 µL of Media A and the cellular suspension was injected subcutaneously into nude mice^54^. Nude mice were injected TAM for 7 days immediately after McSC transplantation. The tumors were harvested at 47 days after transplantation.

### Tumor preparation for RNA extraction and bulk RNA-seq

Tumors from transplantation assays were collected in Media A and minced finely with a scalpel. Tumors were dissociated by agitation in Media A with 0.35% Collagenase I (Worthington) for 1 h at 37 °C, followed by filtering through 100 μm cell strainer to obtain single cell suspension. Tomato^+^ cells were isolated by cell sorting on Beckman Coulter MoFlo cell sorter or Sony SY3200 cell sorter. RNA was extracted from the sorted cells using RNeasy plus micro kit (Qiagen). Purified RNA samples were provided to Genome Technology Center at NYU Langone Medical Center for quality control and sequencing. Paired-end 50 bps raw reads were obtained from Illumina Hiseq-2500 sequencer, demultiplexed using Illumina bcl2fastq conversion software (1.8.4) and mapped to mouse genome (NCBI37/mm9) by using Bowtie aligner (0.12.9)^73^. Mapped reads were assigned to Ensemble gene model (Mus_musculus.NCBIM37.67.gtf) with feature count package^74^. To perform statistical analysis for significant differential expressed genes, edgeR (3.4.2)^75^ was used for the data normalization and comparison between experimental and control groups. The *p* value was adjusted with FDR method. Significant genes were selected as FDR < 0.02 and fold change > 4. The Principal Component Analysis (PCA) plot was generated using DESeq2 (1.22.2)^76^. Quality control data of bulk RNA-seq was shown in Supplementary Table 1.

### Single-cell RNA-Seq and data analysis

Tumors were dissociated into single cell suspension as mentioned above. McSC suspension from 3 telogen *Tyr-CreER:R26R-Tomato* mice and tumor cell suspension from 3 tumors derived from transplantation of McSCs from Tyr-CreER:Braf:Pten:Tomato mice and were used to isolate Tomato^+^ cells by cell sorting on Sony SY3200 cell sorter. The sorted suspensions were mixed into one sample of tumor cell suspension and one sample of McSC suspension.

The sorted cellular suspensions were loaded on a 10x Genomics Chromium instrument to generate single-cell gel beads in emulsion (GEMs). Approximately 5000–10,000 cells were loaded per channel. Single-cell RNA-Seq libraries were prepared using the following Single Cell 3′ Reagent Kits v2: Chromium™ Single Cell 3′ Library & Gel Bead Kit v2, PN-120237; Single Cell 3′ Chip Kit v2 PN-120236 and i7 Multiplex Kit PN-120262 (10x Genomics) following the Single Cell 3′ Reagent Kits v2 User Guide (Manual Part # CG00052 Rev A)^77^. Libraries were run on an Illumina HiSeq 4000 as 2 × 150 paired-end reads, one full lane per sample. Sequencing results were demultiplexed and converted to FASTQ format using Illumina bcl2fastq software. The Cell Ranger Single-Cell Software Suite was used to perform sample demultiplexing, barcode processing, and single-cell 3′ gene counting. The cDNA insert was aligned to the mm10/GRCm38 reference genome. Only confidently mapped, non-PCR duplicates with valid barcodes and UMIs were used to generate the gene-barcode matrix. Further analysis and visualization was performed using Seurat (v2.3.4), an R package containing implementations of commonly used single-cell analytical techniques, including the identification of highly variable genes, dimensionality reduction, standard unsupervised clustering algorithms, and the discovery of differentially expressed genes and markers^78^.

The Seurat object was generated from digital gene expression matrices. The parameter of Filtercells is nGene (200–1000) for McSC, nGene (200–5000) for tumor and percentage of mitochondria genes (0 to 0.1). In the standard pre-processing workflow of Seurat, we selected 4614 highly variable genes for following PCA. Then we performed cell cluster and t-SNE. Ten principal components were used in cell cluster with the resolution parameter set at 0.2, resulting in 7 clusters. We excluded two small clusters that are enriched in keratinocytes and macrophages and used the remaining cells to perform variable gene identification (4834 genes), PCA, cell clustering and t-SNE. Nine principal components were used in cell cluster with the resolution parameter set at 0.33. For pseudo-temporal analysis, digital gene expression matrices with annotations from Seurat were analyzed by Monocle v2.10.1^79^. The top 1, 500 genes with highest dispersion (variation/mean) were used to construct the pseudo-temporal trajectory.

### Gene Set Enrichment Analysis (GSEA)

For the bulk RNA-seq GSEA (Fig. 5f), genes differentially expressed between SC-derived tumor cells and non-transformed McSCs were rank-ordered from high to low based on their fold change. For single cell RNA-seq GSEA (Table 1), genes enriched in each of melanoma cell clusters (clusters 2–5) in comparison compared to McSCs (cluster 1) were rank-ordered based on fold change. For single cell RNA-seq GSEA (Fig. 6c–e), indicated melanoma clusters were compared to each other and their differentially expressed genes were rank-ordered based on fold change. We then queried these pre-ranked gene lists for their enrichment in 4 annotated gene sets acquired from The Molecular Signature Database (MSigDB): GO_CELL_MORPGOGENESIS_INVOLVED_IN_NEURON_DIFFERENTIATION, KEGG_CELL_CYCLE, KEGG_ECM_RECEPTOR_INTERACTION, and LEE_NEURAL_CREST_STEM_CELL_UP^57^, using the preranked GSEA analysis tool^80^. FDR q-value < 0.25 was deemed significant.

### Quantification and statistical analyses

The measurement of quantifications can be found in y-axis of dot plots in the figure and in figure legends. The statistical details of each dot plot can be found in the figure (n number, P value). The exact meaning of n number is described in the corresponding figure legend. Pairwise comparisons between two groups were performed by two-sided unpaired statistical analysis using Student’s *t* test. Statistical significances were considered significant if *P* < 0.05. The exact *P* value is labeled in the figure. Experimental data are demonstrated as the mean ± standard deviation (s.d.). Statistical analysis and plotting was done using Microsoft Excel and Graphpad Prism.

### Reporting Summary

Further information on research design is available in the Nature Research Reporting Summary linked to this article.

## Supplementary information

Supplementary Information

Description of Additional Supplementary Files

Supplementary Data 1

Reporting Summary

Source Data

## Data Availability

All RNA-seq data reported in this paper are deposited in NCBI Gene Expression Omnibus(GEO) database. The accession numbers are GSE96966 for bulk RNA-seq and GSE113502 [https://www.ncbi.nlm.nih.gov/geo/query/acc.cgi] for single cell RNA-seq. The source data underlying all quantifications in Figs. 1–5 and Supplementary Figs. 1–5, 8 and 12 are provided as a Source Data file. All the other data supporting the findings of this study are available within the article and its supplementary information files and from the corresponding author upon reasonable request. A reporting summary for this article is available as a Supplementary Information file.

## Code availability

All the bioinformatics analyses were performed using previously published packages with publicly available codes. Cell Ranger Single-Cell Software (https://support.10xgenomics.com/single-cell-gene-expression/software/pipelines/latest/what-is-cell-ranger); Seurat (https://satijalab.org/seurat/); Monocle (http://cole-trapnell-lab.github.io/monocle-release/); GSEA Broad Institute (https://software.broadinstitute.org/gsea/index.jsp).
