# Supplementary material for: A novel mouse model demonstrates that oncogenic melanocyte stem cells engender melanoma resembling human disease
Source: Nat Commun. 2019 Nov 4;10:5023. doi: 10.1038/s41467-019-12733-1 (PMC6828673; doi:10.1038/s41467-019-12733-1)
Supplement: Supplementary file 1 — Supplementary Information [file 41467_2019_12733_MOESM1_ESM.pdf]

Supplementary Information

A novel mouse model demonstrates that oncogenic melanocyte stem cells engender melanoma resembling human disease

Sun et al.

Supplementary Figures

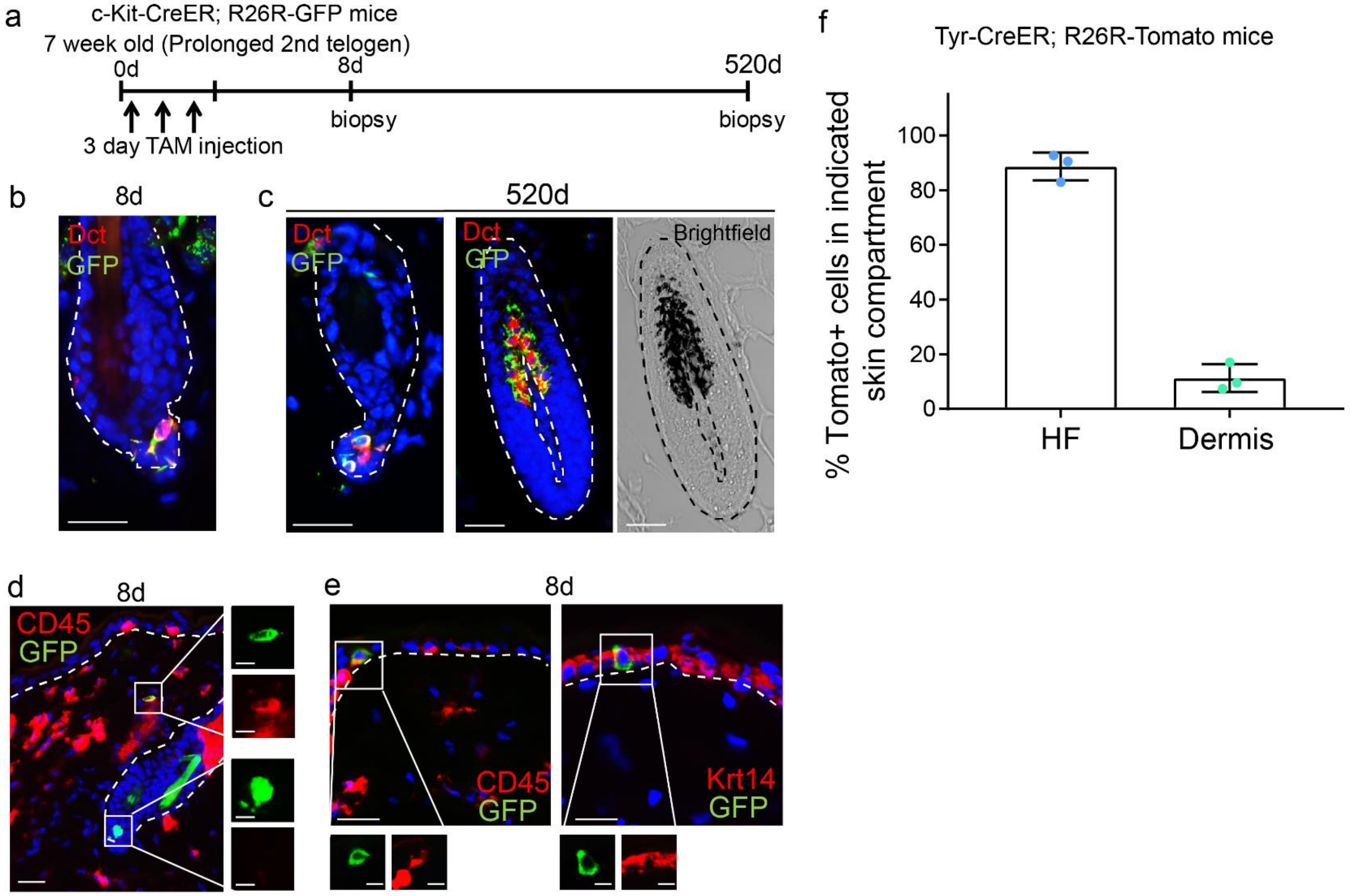

Supplementary Figure 1. The *c-Kit* promoter targets McSCs in the HF. a Schematic showing tamoxifen (TAM) treatment and analysis regimen for (**b-e**). **b** Immunofluorescence for GFP (green) and Dct (red) at 8 days. **c** Immunofluorescence for GFP (green) and Dct (red) in hair follicle bulge (left), bulb (middle) and corresponding brightfield image of bulb (right) at 520 days. The bulb contains mature melanocytes that are producing pigment. **d** Immunofluorescence for GFP (green) and CD45 (red) at 8 days. **e** Immunofluorescence for GFP (green) and CD45, Keratin14 (red) showing the interfollicular epidermis at 8 days. **f** *Tyr-CreER; R26R-Tomato* mice were treated with TAM for 3 days and tissue analyzed at day 8 (see panel **a**). Quantification of the percentage of Tomato positive cells located in the hair follicle (HF) or dermal compartment (mean ± s.d., n= 3 independent mice with 10 different areas of skin each). Dashed line outlines the boundary of epithelium and dermis. Scale bar, 25 μm. Scale bar of separate channel images in (**d**) and (**e**), 10 μm. Source data are provided as a Source Data file.

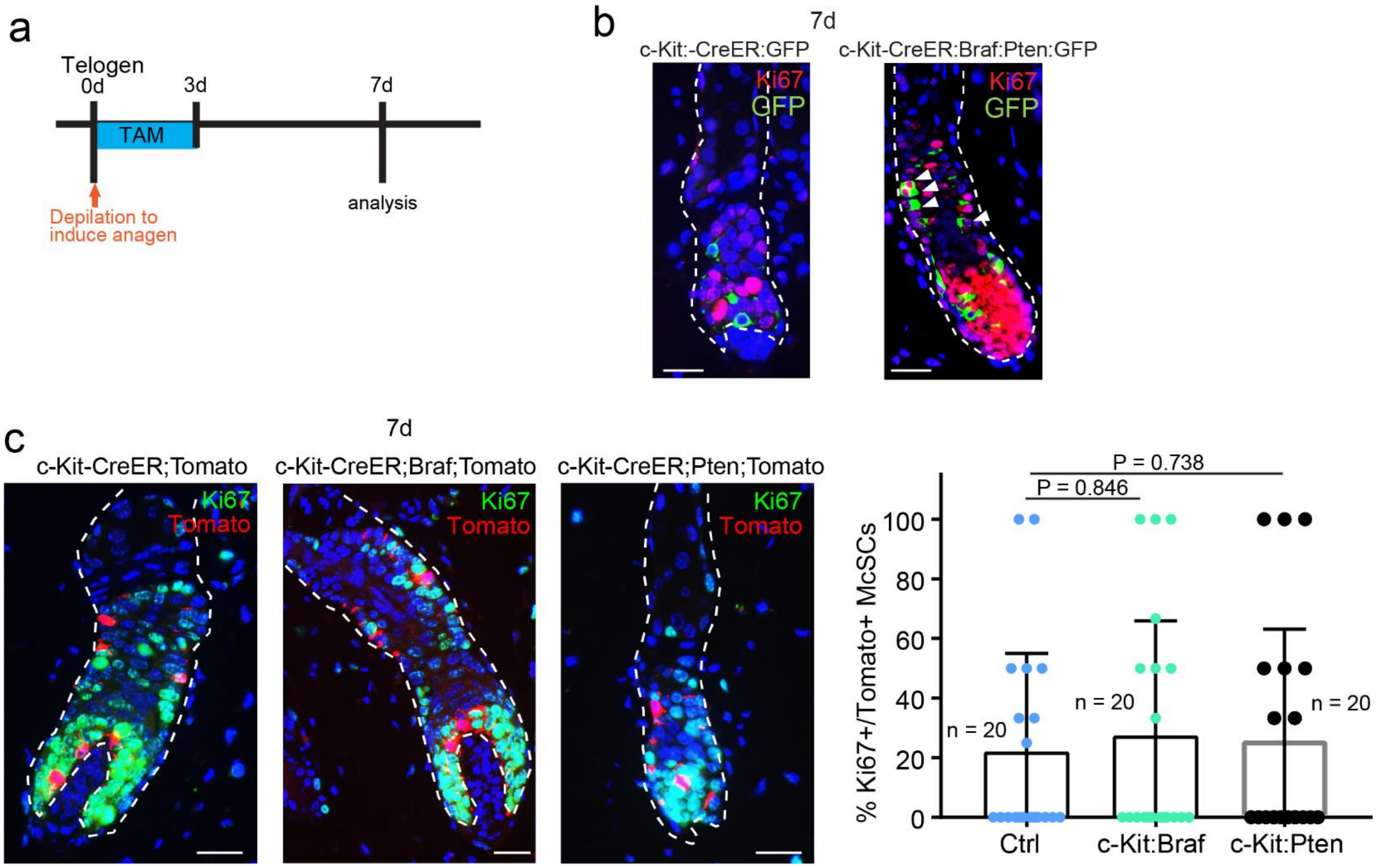

Supplementary Figure 2. *Braf V600E* expression or *Pten* loss alone is not sufficient to transform McSCs. a Schematic showing TAM treatment and analysis regimen for (**b, c**). **b** Immunofluorescence for Ki67 (red) and GFP (green) of c-Kit-CreER: GFP and c-Kit-CreER:Braf:Pten:GFP mouse skin at 7 days. **c** Immunofluorescence for Tomato (red) and Ki67 (green) of *c-Kit-CreER; R26R-Tomato*, *c-Kit-CreER; Braf CA/+; R26R-Tomato* and *c-Kit-CreER; Pten fl/fl; R26R-Tomato* mouse skin at 7 days. Rightmost panel shows quantification of the percentage of Ki67+ cells among Tomato+ McSCs (mean± s.d., n = 20 individual hair follicles of 2 independent mice). Scale bar, 25 μm. Source data are provided as a Source Data file.

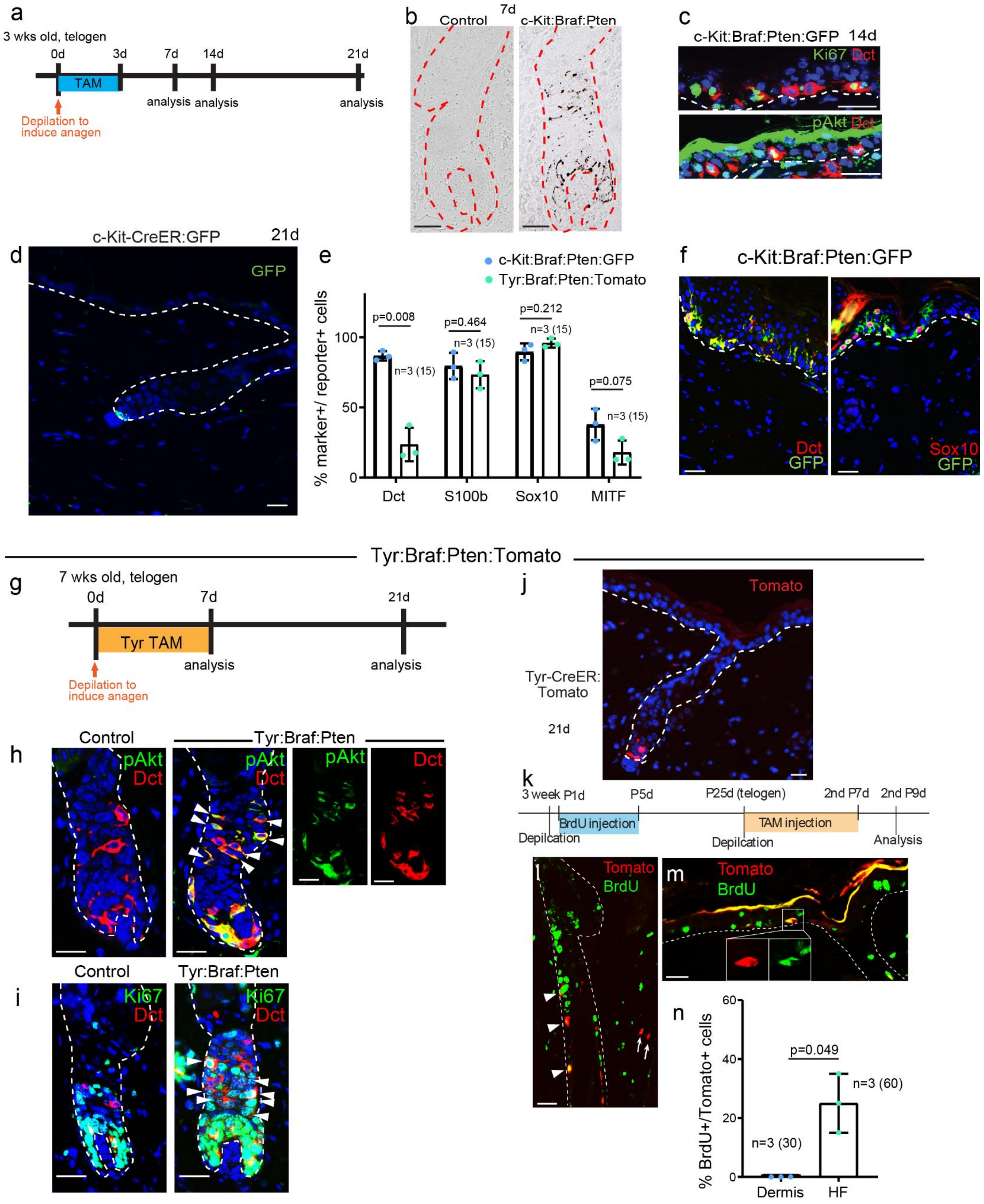
a
3 wks old, telogen
0d
3d
7d
14d
21d
TAM
analysis
analysis
analysis
Depilation to
induce anagen
b
Control
7d
c-Kit:Braf:Pten
c
c-Kit:Braf:Pten:GFP
14d
Ki67
Dct
pAkt
Dct
d
c-Kit-CreER:GFP
21d
GFP
e
c-Kit:Braf:Pten:GFP
Tyr:Braf:Pten:Tomato
% marker+/ reporter+ cells
p=0.008
p=0.464
p=0.212
p=0.075
n=3 (15)
n=3 (15)
n=3 (15)
n=3 (15)
100
50
0
Dct
S100b
Sox10
MITF
f
c-Kit:Braf:Pten:GFP
Dct
GFP
Sox10
GFP
Tyr:Braf:Pten:Tomato
g
7 wks old, telogen
0d
7d
21d
Tyr TAM
analysis
analysis
Depilation to
induce anagen
j
Tomato
Tyr-CreER:
Tomato
21d
h
Control
Tyr:Braf:Pten
pAkt
Dct
pAkt
Dct
pAkt
Dct
k
3 week
P1d
P5d
P25d (telogen)
2nd P7d
2nd P9d
BrdU injection
TAM injection
Depilcation
Depilcation
Analysis
l
Tomato
BrdU
m
Tomato
BrdU
i
Control
Tyr:Braf:Pten
Ki67
Dct
Ki67
Dct
n
% BrdU+/Tomato+ cells
60
40
20
0
p=0.049
n=3 (60)
n=3 (30)
Dermis
HF

Supplementary Figure 3. McSCs give rise to epidermal melanoma. a Schematic showing TAM treatment and analysis regimen for (**b-f**). **b** Brightfield images of control (left) and c-Kit-CreER:Braf:Pten:GFP (right) mice skin at 7 days. **c** Immunofluorescence for Dct (red) and Ki67, pAKT (green) in epidermis of c-Kit-CreER:Braf:Pten:GFP mice skin at 14 days. **d** Immunofluorescence for GFP in c-Kit-CreER:GFP control mouse at 21 days. **e** Quantification of the percentage of Dct, S100b, Sox10 and MITF positive cells among reporter positive cells in c-Kit-CreER:Braf:Pten:GFP and Tyr-CreER:Braf:Pten:Tomato mice at 21day (mean ± s.d., n = 3 independent mice, total number of skin areas analyzed is indicated in parenthesis in each group). **f** Immunofluorescence for GFP (green) and Dct, Sox10 (red) in c-Kit-CreER:Braf:Pten:GFP mice skin at 14 days. **g** Schematic showing TAM treatment and analysis regimen for (**h-j**). TAM was injected for 3 days in (**j**). **h** Immunofluorescence for Dct (red) and pAKT (green) in control (left) and Tyr:Braf:Pten (middle) skin at 7 days after initial TAM treatment. Separate channels images for the Tyr:Braf:Pten panel show distinct regions of overlap, demonstrating pAKT signals in Dct^+ McSCs. **i** Immunofluorescence for Dct (red) and Ki67 (green) in control (left) and Tyr:Braf:Pten (middle) skin at 7 days after initial TAM. **j** Immunofluorescence for Tomato in Tyr-CreER: Tomato control mouse at 21 days. **k** Schematic showing TAM treatment and analysis regimen for (**l-n**). P“x”d: “x” days after depilation. **l, m** Immunofluorescence for Tomato (red) and BrdU (green) at 2nd P9d. **n** Quantification of $BrdU^+$ cells in $Tomato^+$ cells in the dermis and hair follicle at 2nd P9d (mean ± s.d., n = 3 independent mice, total number of skin areas or HFs analyzed is indicated in parenthesis in each group). Dashed line outlines the boundary of epithelium and dermis. Scale bar, 25 μm. Arrowheads, follicular McSCs. Source data are provided as a Source Data file.

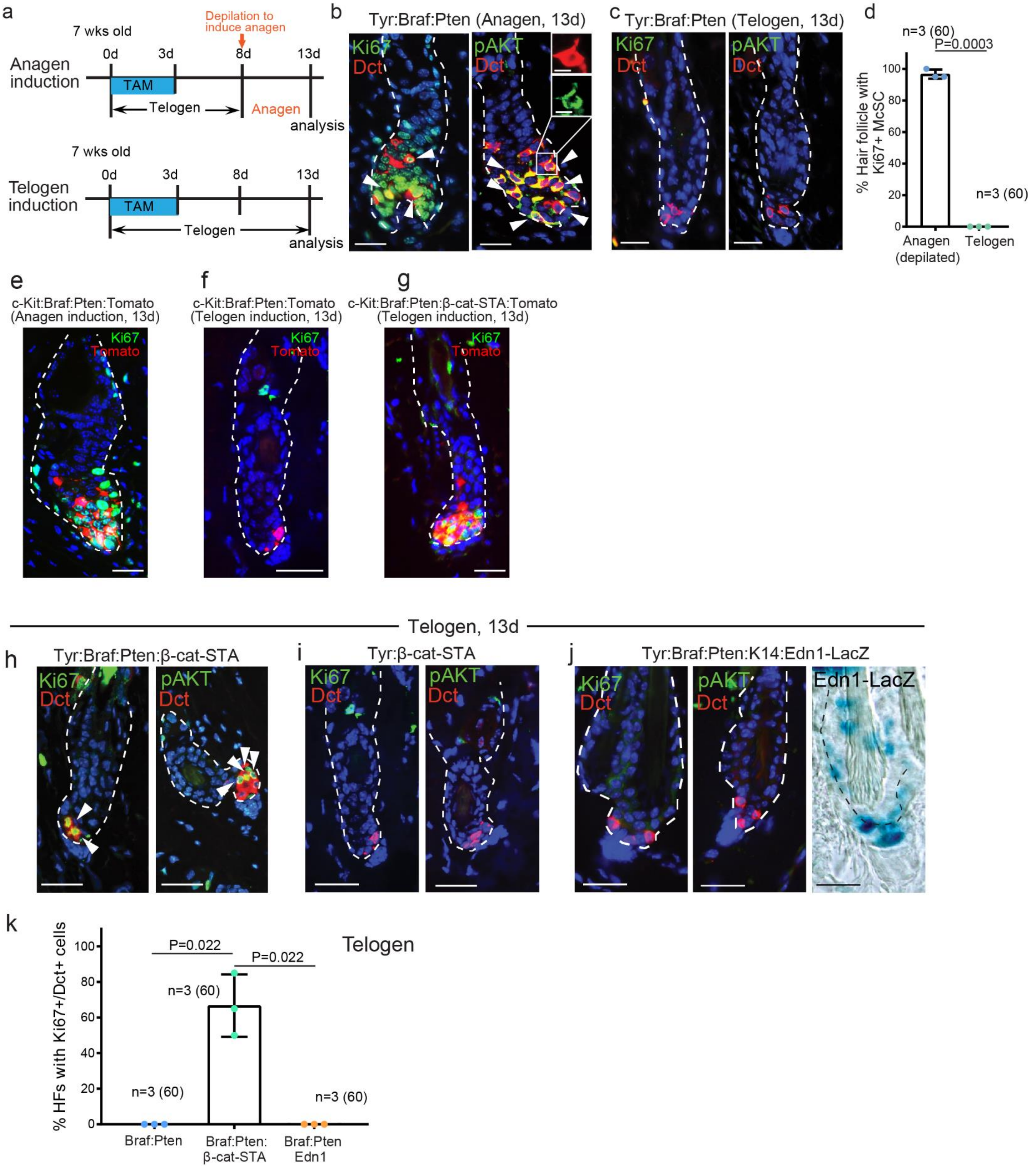

Supplementary Figure 4. Wnt promotes McSC transformation in telogen. a Schematic showing TAM treatment and analysis regimen. Top panel is experimental scheme for (**b**) and (**e**); bottom panel is experimental scheme for (**c, f-j**). Mice in (**e-g**) were treated for TAM for 3 days. Mice in (**j**) were also treated with 1g/KG

Doxycycline diet from 0d-13d. **b, c** Immunofluorescence for Dct (red) and Ki67, pAKT (green) in Tyr-CreER:Braf:Pten mouse skin with anagen induction (**b**) and telogen induction (**c**). Inset in (**b**) shows separate channel images with higher magnification of boxed area. **d** Dot plot showing percentage of HFs with $Ki67^{+}Dct^{+}$ McSCs (mean ± s.d., n = 3 independent mice, total number of HFs analyzed is indicated in parenthesis in each group). **e-g** Immunofluorescence for Tomato (red) and Ki67 (green) in c-Kit-CreER:Braf:Pten:Tomato mice with anagen induction (**e**), telogen induction (**f**), and c-Kit-CreER:Braf:Pten:β-cat-STA:Tomato mice with telogen induction (**g**). **h-j** Immunofluorescence for Dct (red) and Ki67, pAKT (green) in Tyr-CreER:Braf:Pten:β-cat-STA (**h**), Tyr-CreER:β-cat-STA (**i**) and Tyr-CreER:Braf:Pten:K14-rtTA:tetO-Edn1-LacZ (**j**) mouse skin with telogen induction. The right panel in (**j**) is image of X-gal staining showing expression of Edn1. **k** Bar graph showing percentage of HFs with $Ki67^{+}Dct^{+}$ McSCs (mean ± s.d., n = 3 independent mice, total number of HFs analyzed is indicated in parenthesis in each group). Dashed line outlines the boundary of epithelium and dermis. Scale bar, 25 μm. Scale bar of separate channel images in (**b**), 10 μm Arrowheads, double positive McSCs. Source data are provided as a Source Data file.

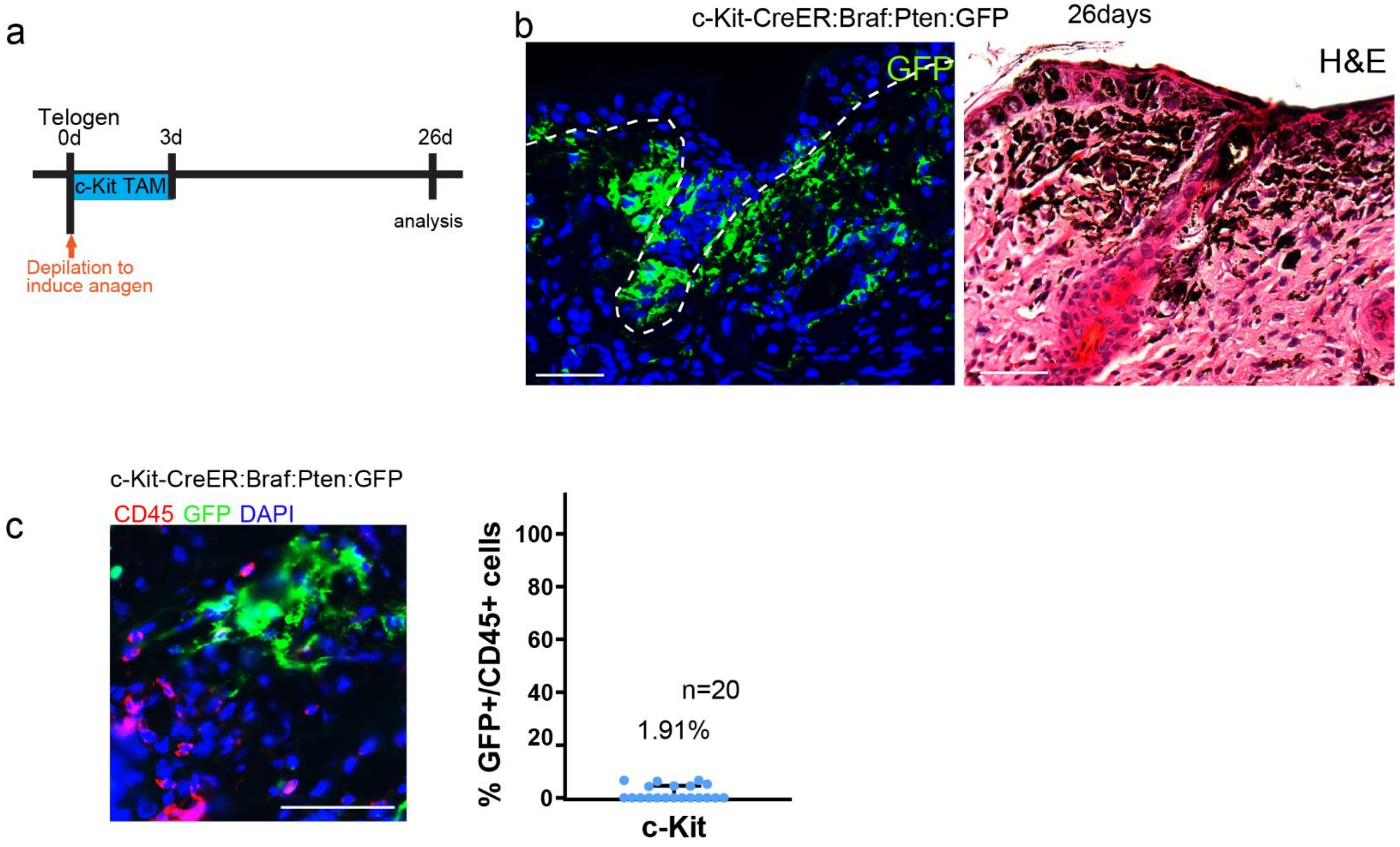

Supplementary Figure 5. *C-Kit* melanoma mice form dermal melanoma without targeting of CD45^{+} cells. **a** Schematic showing TAM treatment and analysis regimen for (**b-d**). **b** Immunofluorescence for GFP (left) and H&E image (right) in skin of c-Kit-CreER:Braf:Pten:GFP mice at 26 days. **c** Immunofluorescence for CD45 (red) and GFP (green) in the dermis of c-Kit-CreER:Braf:Pten:GFP mice at 26 days. Quantification of the percentage of GFP labeled CD45^{+} cells (mean ± s.d.; n= 20 different areas of skin of 2 independent mice) suggests that CD45^{+} hematopoietic lineage cells affected by *c-Kit-CreER* are negligible. Dashed line outlines the boundary of epithelium and dermis. Scale bar, 50 μm. Source data are provided as a Source Data file.

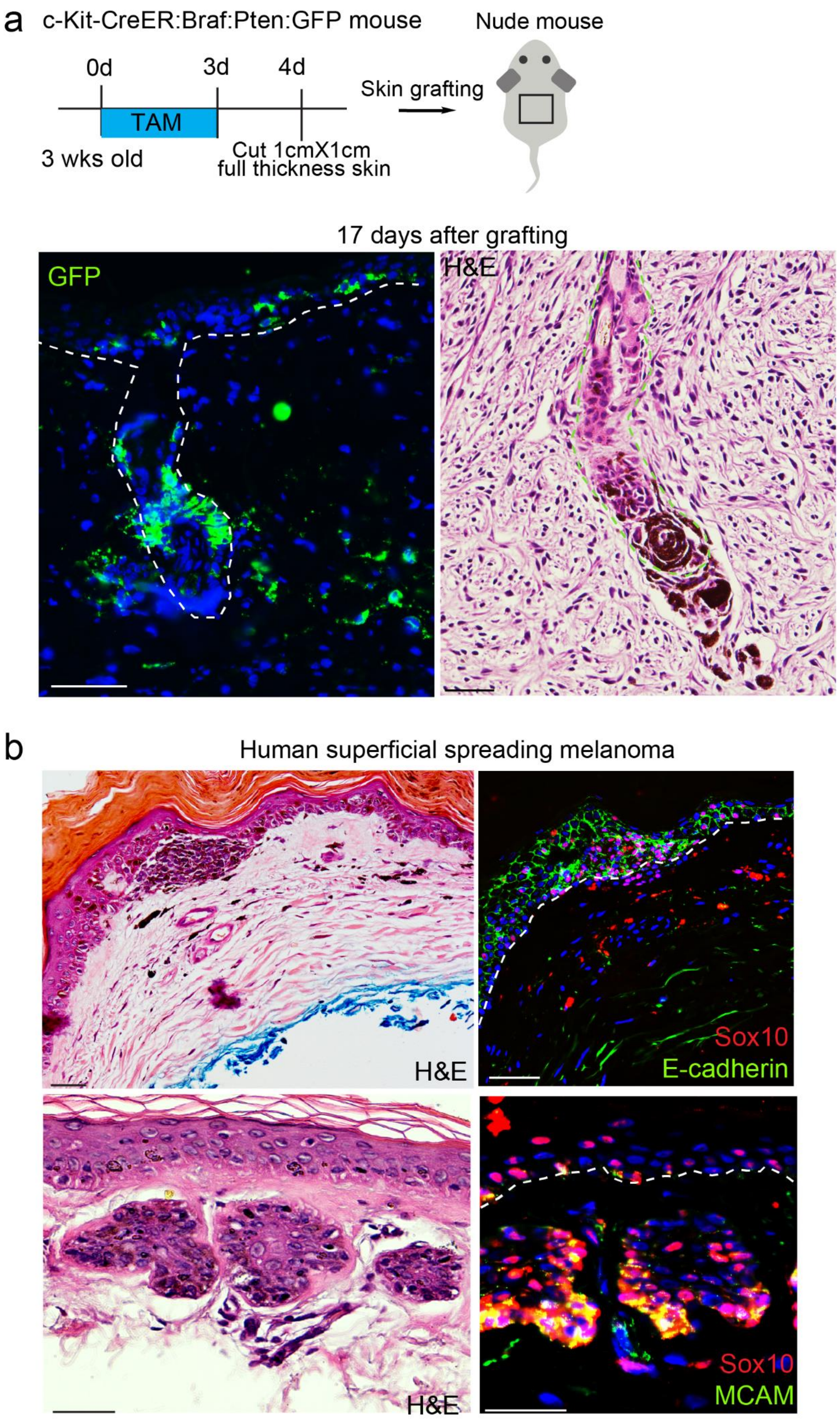
a
c-Kit-CreER:Braf:Pten:GFP mouse
Nude mouse
0d
3d
4d
TAM
Skin grafting
3 wks old
Cut 1cmX1cm
full thickness skin
17 days after grafting
GFP
H&E
b
Human superficial spreading melanoma
H&E
Sox10
E-cadherin
H&E
Sox10
MCAM

Supplementary Figure 6. Epidermal melanoma invades into the dermis. a Schematic showing TAM treatment and skin implantation (Left). Immunofluorescence for GFP at 17 days after skin implantation of c-Kit-CreER:Braf:Pten:GFP mice (middle) and H&E image of identical area (Right). **b** H&E and immunofluorescence for Sox10 (red) and E-cadherin, MCAM (green) in human superficial spreading melanoma. The Immunofluorescence images are low magnification images of Fig. **4e**. The H&E images are areas identical to the immunofluorescence on tissue sections of the same human melanoma samples. Dashed line outlines the boundary of epithelium and dermis. Scale bar, 50 μm.

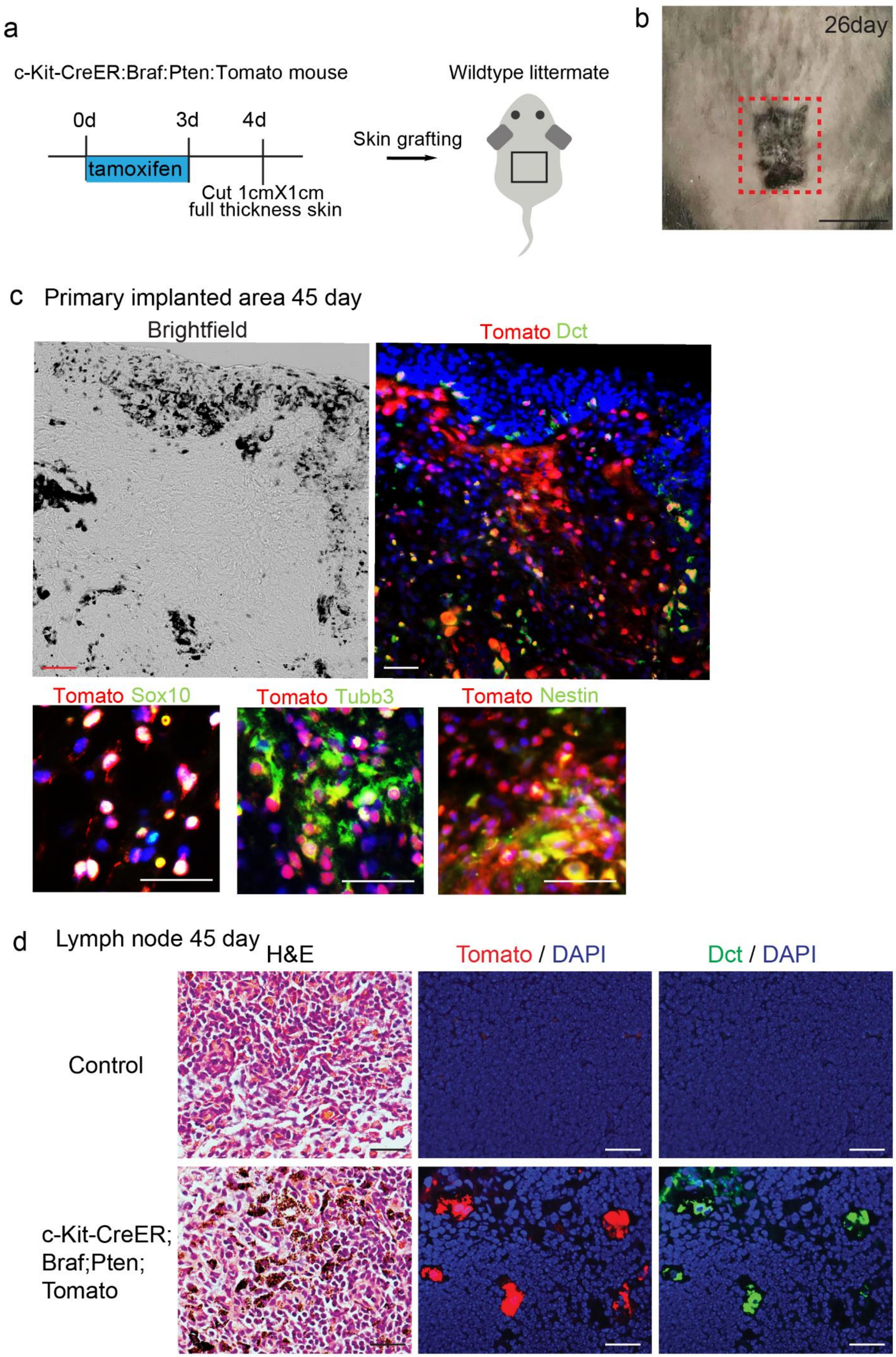
a
c-Kit-CreER:Braf:Pten:Tomato mouse
0d
3d
4d
tamoxifen
Cut 1cmX1cm
full thickness skin
Skin grafting
Wildtype littermate
b
26day
c
Primary implanted area 45 day
Brightfield
Tomato Dct
Tomato Sox10
Tomato Tubb3
Tomato Nestin
d
Lymph node 45 day
H&E
Tomato / DAPI
Dct / DAPI
Control
c-Kit-CreER;
Braf;Pten;
Tomato

Supplementary Figure 7. Grafting skin of c-Kit-CreER:Braf:Pten:Tomato mice onto syngeneic littermates leads to formation of primary melanoma and lymph node metastasis. a Schematic showing TAM treatment and skin implantation onto syngeneic mice for (k-m). **b** Image of implanted skin on the recipient showing formation of pigmented melanoma tumor. **c** Immunofluorescence images for Tomato (red) and Dct, Sox10, Tubb3, Nestin (green) and brightfield images in implanted skin at 45 days after grafting. **d** Immunofluorescence images for Tomato (red), Dct (green) and H&E images in lymph node of recipients of control and c-Kit-CreER:Braf:Pten:Tomato mouse skin at 45 days after grafting. Red dashed line outlines the boundary of implanted skin. Scale bar, 50 μm, except in (**b**) which is 1cm.

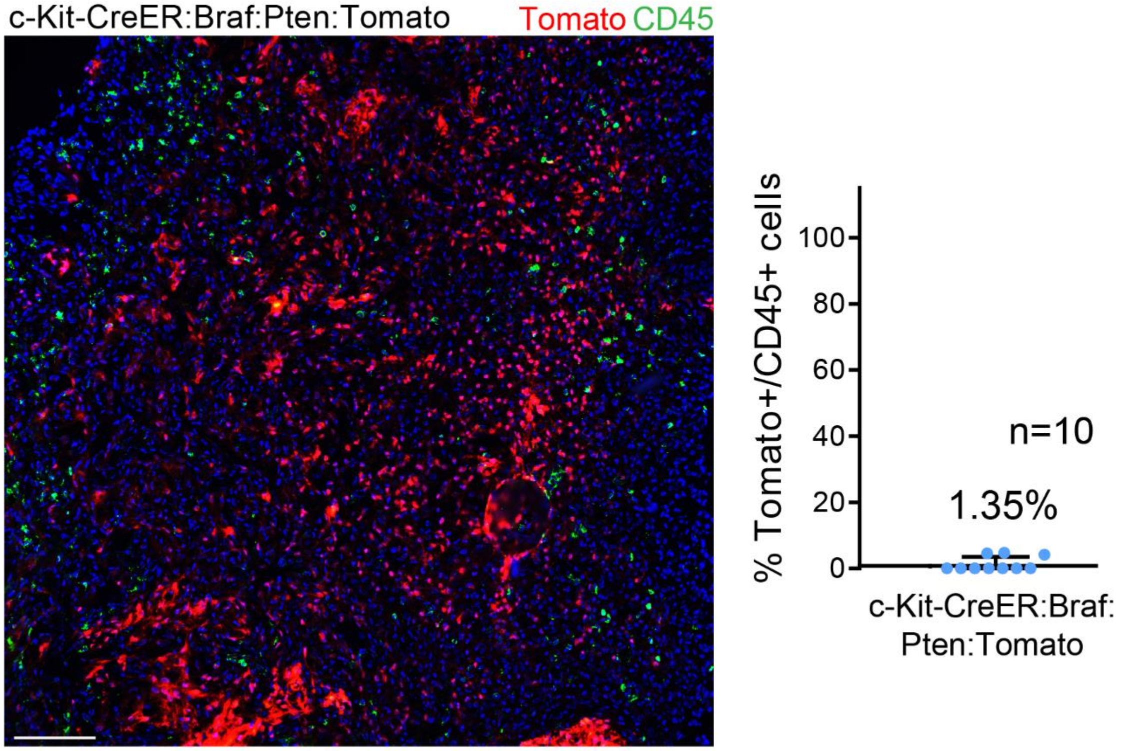

Supplementary Figure 8. Topical 4-hydroxytamoxifen treatment onto *c-Kit* melanoma mice barely targets tumor infiltrating immune cells. Immunofluorescence images for Tomato (red) and CD45 (green) in c-Kit-CreER:Braf:Pten:Tomato mouse skin at 47 days after topical 4HT-TAM treatment. Quantification of the percentage of Tomato reporter labeled CD45+ cells (mean ± s.d.; n= 10 different tumor areas of 3 independent mice) suggests that CD45+ hematopoietic lineage cells affected by *c-Kit-CreER* are negligible. Scale bar, 100 μm. Source data are provided as a Source Data file.

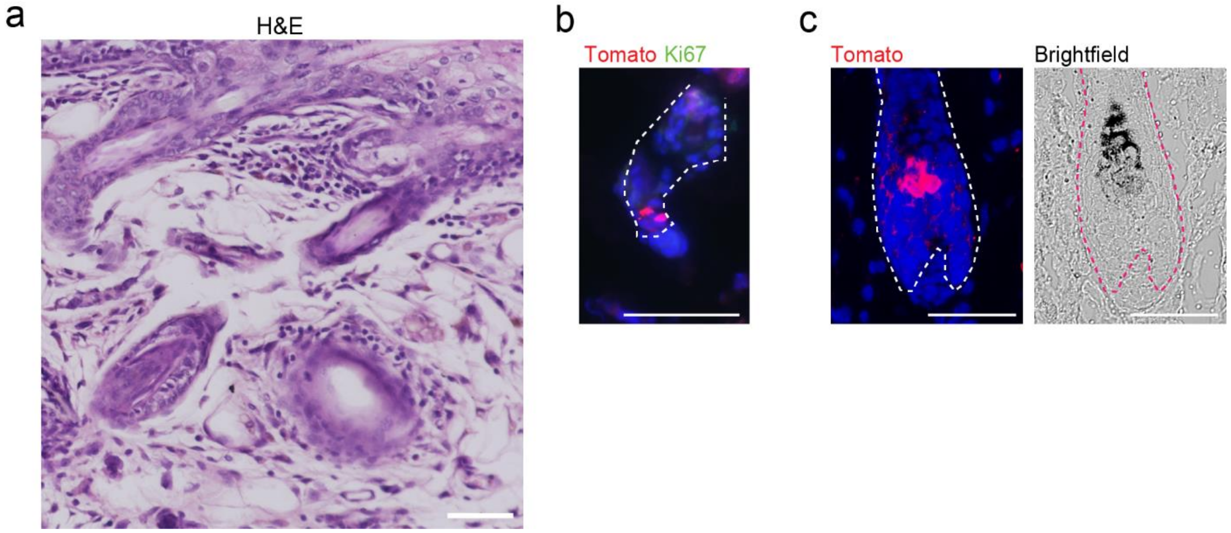

Supplementary Figure 9. Transplanted wildtype McSCs become bulge and pigmented bulb melanocytes instead of forming tumor. a-c McSCs were isolated from the skin of *Tyr-CreER; R26R-Tomato* mice and transplanted with wildtype support cells into immunocompromised nude mice. Nude mice were injected TAM for 7 days to induce transgene expression. H&E image showing the formation of HFs instead of tumor (**a**). Immunofluorescence of Tomato (red) and Ki67 (green) in a HF bulge area (**b**). Immunofluorescence of Tomato (red) and corresponding brightfield images of a HF bulb area (**c**). Dashed line outlines the boundary of epithelium and dermis. Scale bar, 50 μm.

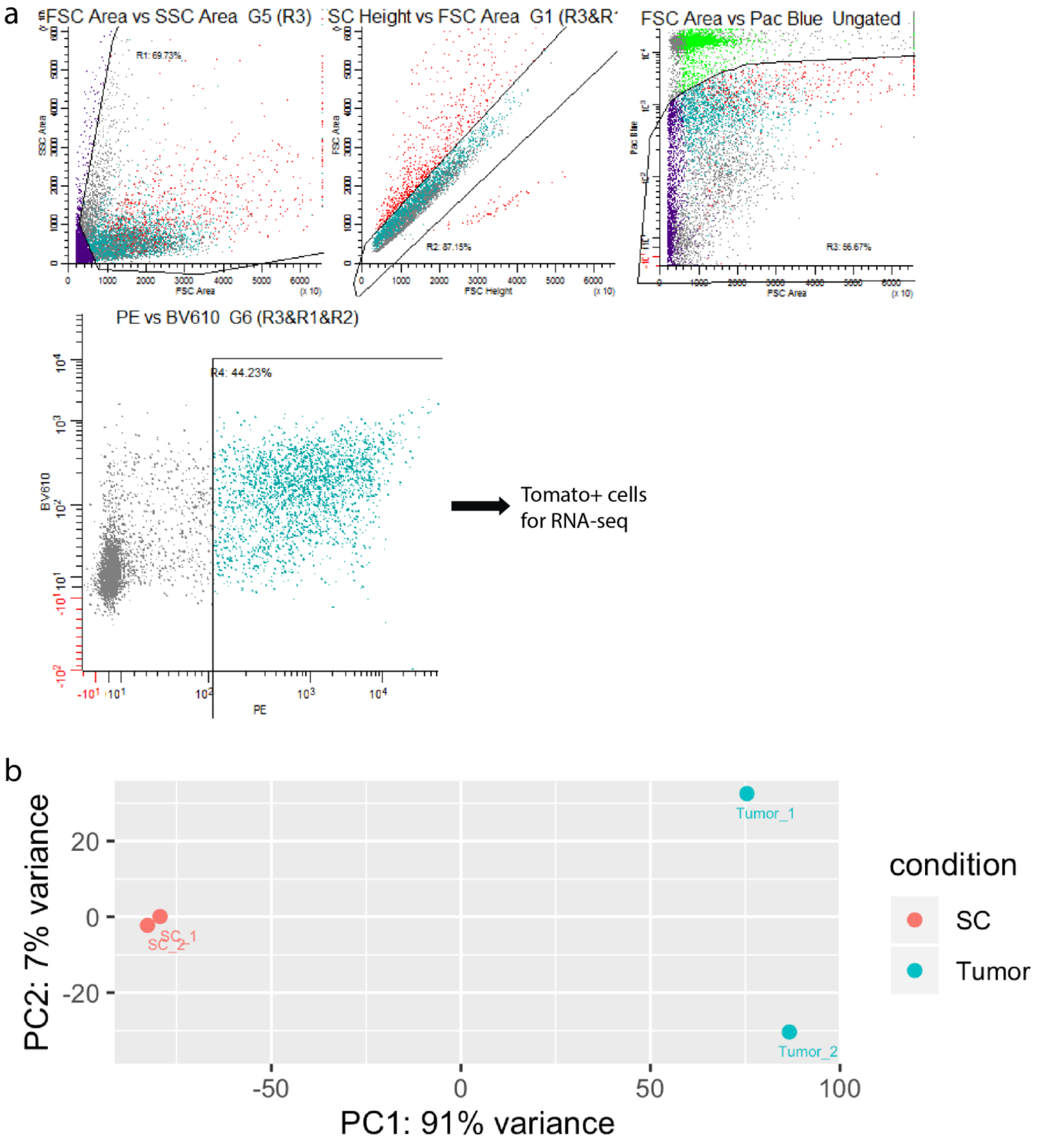

Supplementary Figure 10. Bulk RNA-seq using Tomato+ cells isolated by flow cytometry. a Cell sorting strategy to isolate Tomato+ McSCs and melanoma cells derived from them for bulk and single cell RNA-seq. Forward scatter (FSC) and side scatter (SSC) were performed to exclude cell debris and doublets. Living cells were selected by DAPI exclusion (Pac Blue low). Finally, McSCs or melanoma cells derived from them labeled

by Tomato reporter were isolated as Tomato (PE) + cells and subjected to RNA-seq. This figure shows an experiment of isolation of Tomato+ melanoma cells at 47 days after transplantation of McSCs. The gates shown here are the actual gates used in this experiment. **b** Principal component analysis (PCA) plot of bulk RNA-seq data showing the correlation between SC and tumor cells as well as correlation of biological replicates. The first principal component (PC1, x-axis) specifies the direction with 91% variability in the data, the second component (PC2, y-axis) specifies the direction with 7% variability in the data.

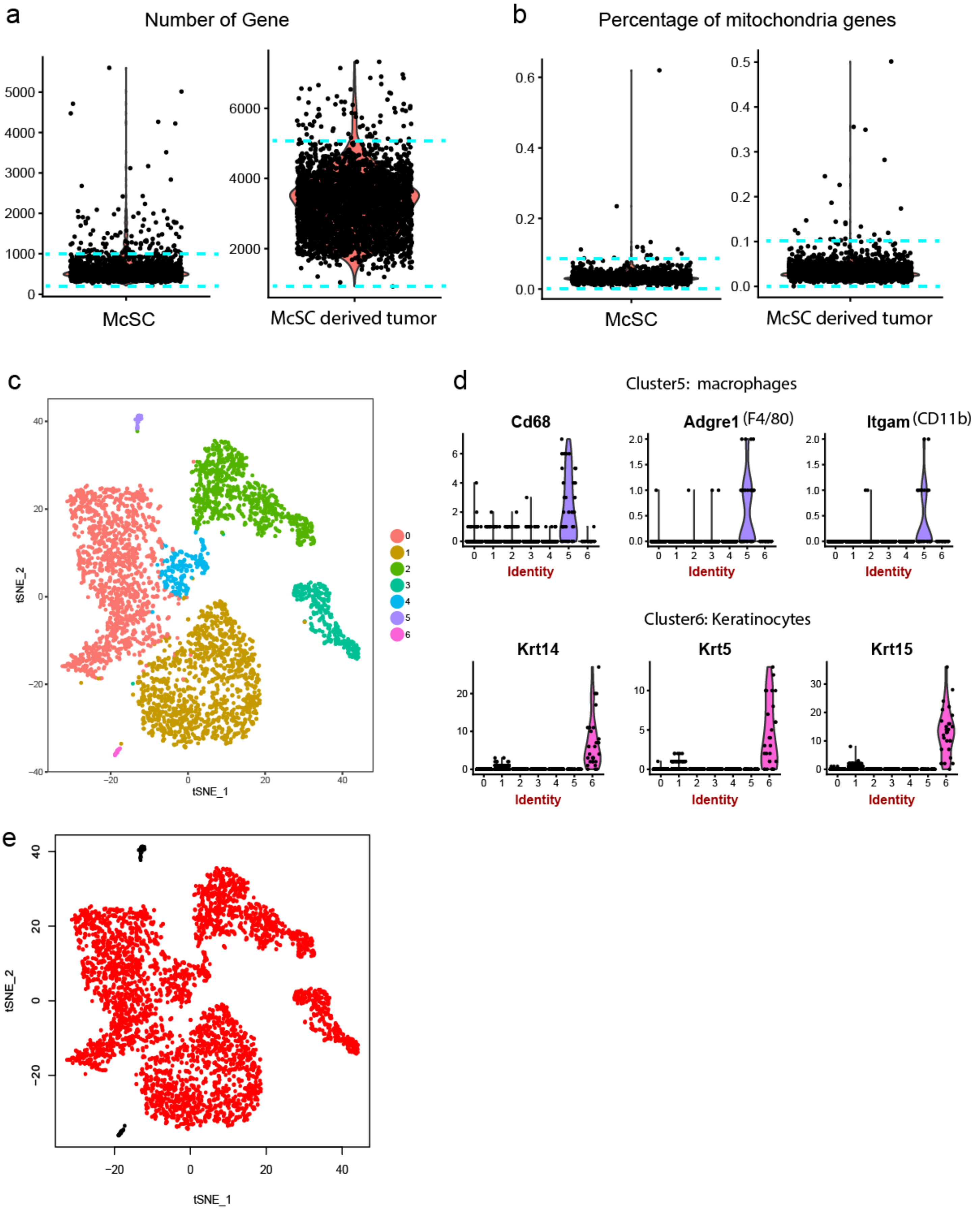

a
Number of Gene
McSC
McSC derived tumor
b
Percentage of mitochondria genes
McSC
McSC derived tumor
c
tSNE_1
tSNE_2
d
Cluster5: macrophages
Cd68
Adgre1 (F4/80)
Itgam (CD11b)
Identity
Cluster6: Keratinocytes
Krt14
Krt5
Krt15
Identity
e
tSNE_1
tSNE_2

Supplementary Figure 11. Quality control and removal of contaminated keratinocytes and macrophages of single cell RNA-seq data set. a Violin plots showing the number of detected genes in each single cell in McSC and McSC derived tumor groups. Dashed cyan lines indicate the cut-off to filter out cells with too low gene expression (less than 200) or too high detected genes (1000 for McSC, 5000 for McSC derived tumor). **b** Violin plots showing the percentage of mitochondria genes in each single cell of McSC and McSC derived tumor. Dashed cyan lines indicate the cut-off to exclude cells with too high mitochondria genes (potentially dying cells, 0 to 0.1 for both groups). **c** t-SNE plot showing the segregation of all sequenced single cells into 7 clusters based on unbiased clustering. **d** Violin plots show the distribution of macrophage and keratinocyte markers in the 7 clusters. Cluster 5 is enriched for macrophage markers such as Cd68, Adgre1 and Itgam. Cluster 6 is enriched for keratinocyte markers such as Krt14, Krt5 and Krt15. **e** t-SNE plot showing the removal of clusters 5 and 6 (black cells) from the subsequent analyses of McSCs and melanoma cells (red cells).

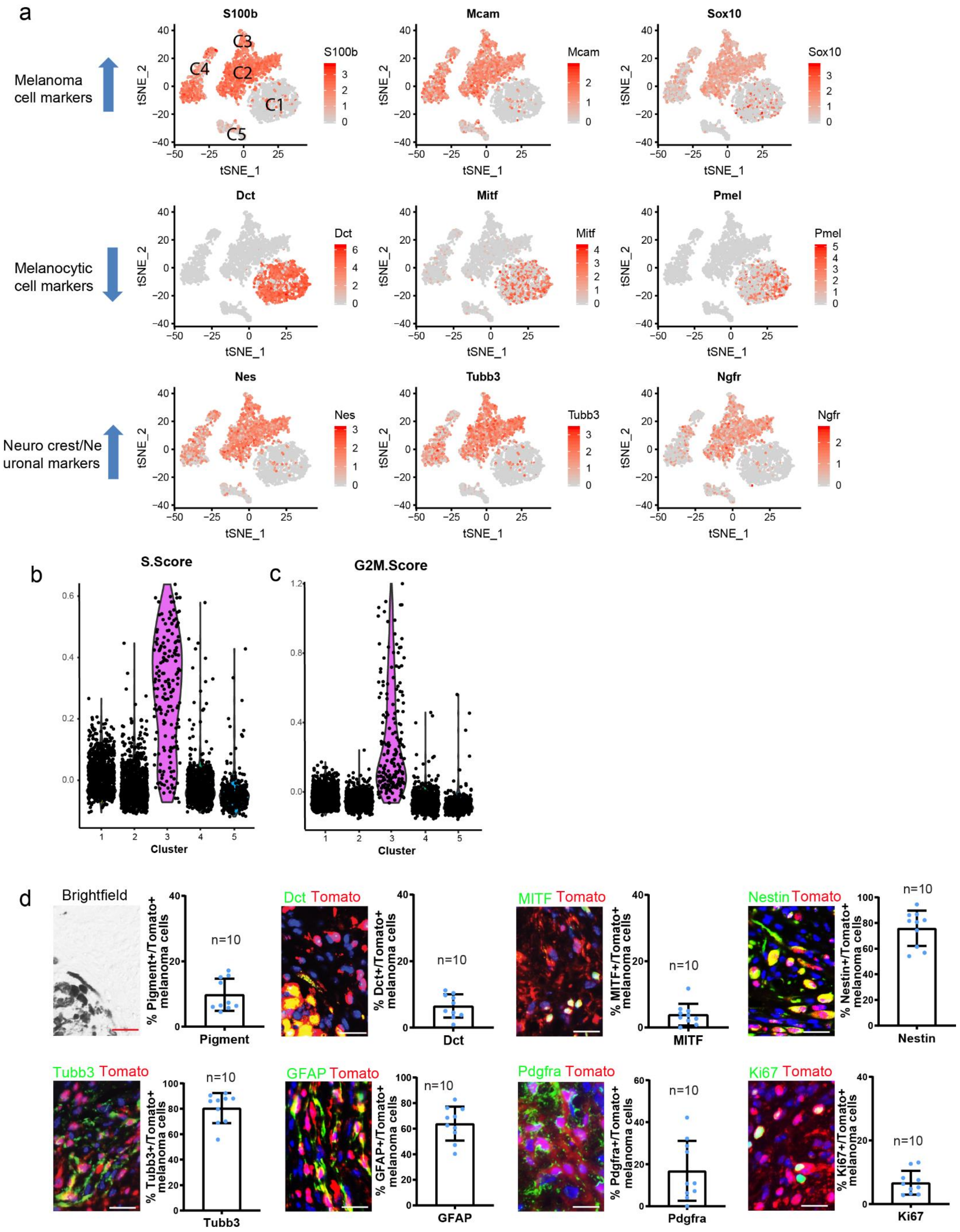
a
Melanoma cell markers
Melanocytic cell markers
Neuro crest/Neuronal markers
S100b
Mcam
Sox10
Dct
Mitf
Pmel
Nes
Tubb3
Ngfr
C1
C2
C3
C4
C5
tSNE_1
tSNE_2
b
S.Score
c
G2M.Score
Cluster
d
Brightfield
% Pigment+/Tomato+ melanoma cells
Pigment
Dct Tomato
% Dct+/Tomato+ melanoma cells
Dct
MITF Tomato
% MITF+/Tomato+ melanoma cells
MITF
Nestin Tomato
% Nestin+/Tomato+ melanoma cells
Nestin
Tubb3 Tomato
% Tubb3+/Tomato+ melanoma cells
Tubb3
GFAP Tomato
% GFAP+/Tomato+ melanoma cells
GFAP
Pdgfra Tomato
% Pdgfra+/Tomato+ melanoma cells
Pdgfra
Ki67 Tomato
% Ki67+/Tomato+ melanoma cells
Ki67
n=10

Supplementary Figure 12. McSC derived tumors heterogeneously express melanocytic, neuronal, proliferative and mesenchymal markers. a FeaturePlot showing the expression of signature genes detected in single cell RNA-seq. The color scales illustrates the scaled expression of indicated genes. **b, c** Violin plots showing the S.Score (**b**) and G2M.Score (**c**) of single cells in each of the 5 clusters. **d** Immunofluorescence images for Tomato (red) and Dct, MITF, Nestin, GFAP, Tubb3, Pdgfra, Ki67 (green) and brightfield image from tumors derived from McSCs of c-Kit-CreER:Braf:Pten:Tomato mice at 47 days following transplantation. Quantification of the percentage of Tomato+ tumor cells positive for indicated markers at 47 days after tamoxifen induction (mean ± s.d.; n= 10 distinct tumors from 7 mice; 4 tumors were from the transplantation of McSCs of c-Kit:Braf:Pten:Tomato mice onto 2 nude mice; 3 tumors were from the ttransplantation of McSCs of Tyr:Braf:Pten:Tomato mice onto 2 nude mice; 3 tumors were from in vivo topical 4HT-TAM treatment of 3 c-Kit:Braf:Pten:Tomato mice). These data showed that about 70% tumor cells express neuronal markers Nestin , Tubb3 and GFAP. Other markers are expressed by fewer cells. These data are consistent with our single cell RNA-seq results which show that many more melanoma cells express neuronal markers than melanocytic, mesenchymal or proliferative markers at this time point. Scale bar, 20 μm. Source data are provided as a Source Data file.

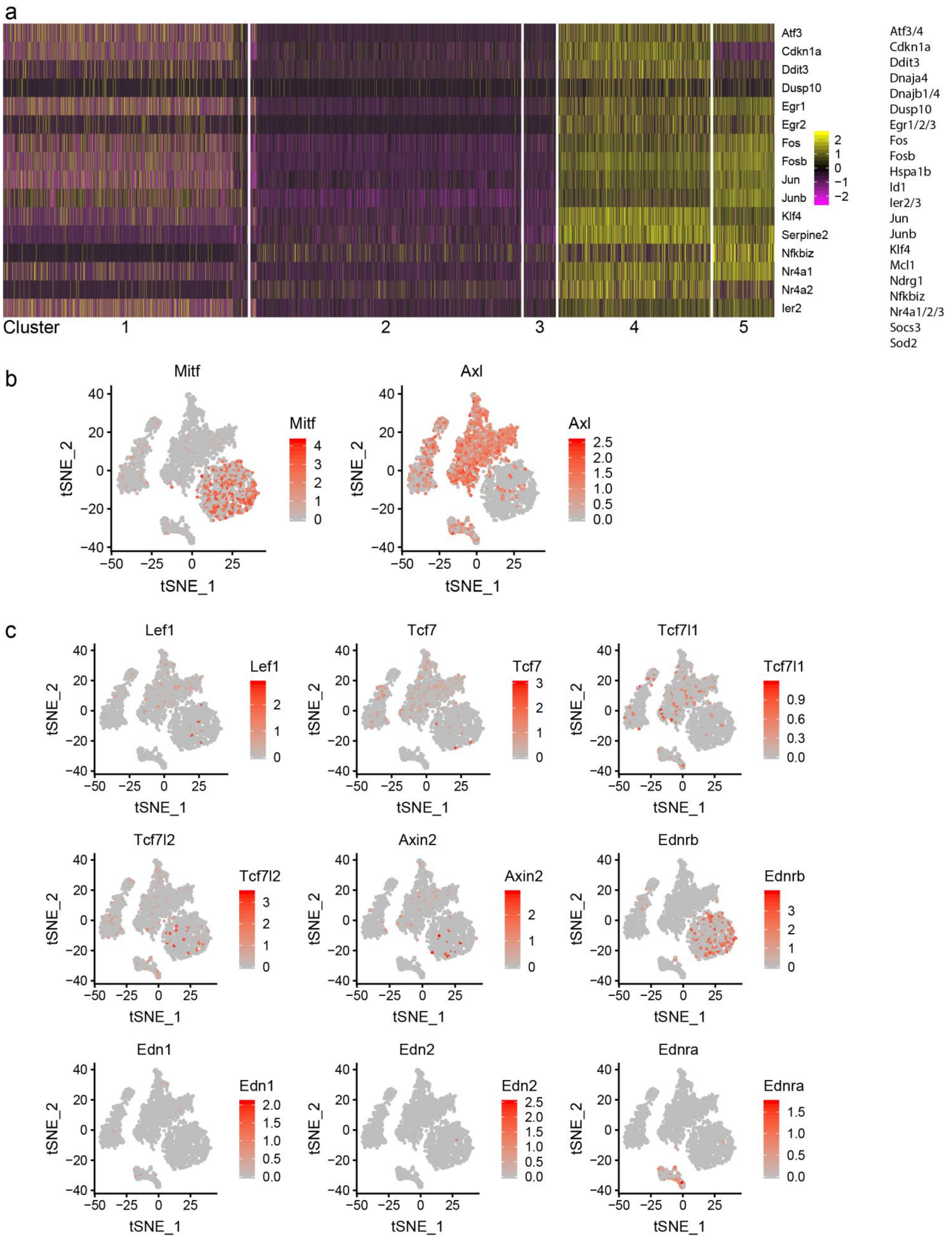
a
Atf3
Cdkn1a
Ddit3
Dusp10
Egr1
Egr2
Fos
Fosb
Jun
Junb
Klf4
Serpine2
Nfkbiz
Nr4a1
Nr4a2
Ier2
Cluster 1 2 3 4 5
Atf3/4
Cdkn1a
Ddit3
Dnaja4
Dnajb1/4
Dusp10
Egr1/2/3
Fos
Fosb
Hspa1b
Id1
Ier2/3
Jun
Junb
Klf4
Mcl1
Ndrg1
Nfkbiz
Nr4a1/2/3
Socs3
Sod2
b
Mitf
Axl
tSNE_1
tSNE_2
c
Lef1
Tcf7
Tcf7l1
Tcf7l2
Axin2
Ednrb
Edn1
Edn2
Ednra

Supplementary Figure 13. Heatmap and feature plots of gene expression in single cells. a Heatmap of scaled expression of a set of enriched genes in Clusters 4 and 5. Right panel is a comparative published gene set enriched in single cell RNA-seq of human metastatic melanoma[1]. **b** Feature plots showing the distribution of *MITF*-high and *Axl*-high cells in each cluster. **c** Feature plots showing the expression of Wnt and Edn related genes in single cells. The color scales in **a-c** illustrates the scaled expression of indicated genes.

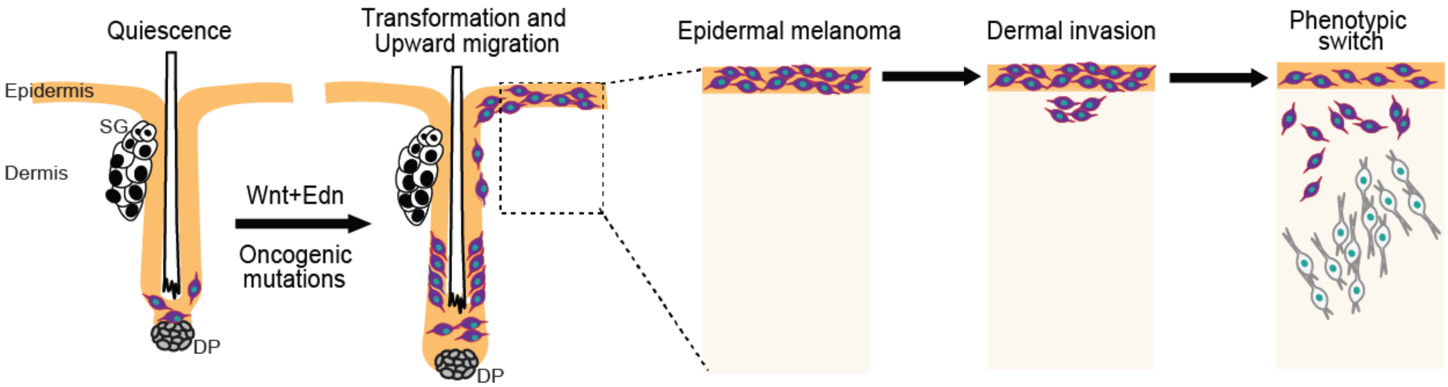

Supplementary Figure 14. Graphic summary. Homeostatic regenerative signals synergize with oncogenic mutations in McSCs, leading to the formation of epidermal melanoma, which then invades into the dermis to finally establish heterogeneous melanoma via phenotypic switch.

Supplementary Tables

Supplementary Table 1. Quality control data of bulk RNA-seq. All data were generated by Illumina bcl2fastq conversion software except for the percent mapped reads, which was generated by Bowtie read aligner.

Sample ID	Total read count	% Perfect Index Reads	% One Mismatch Reads (Index)	% of >= Q30 Bases (PF)	Mean Quality Score (PF)	% mapped reads
SC_1	28,121,156	98.77	1.23	95.92	35.69	90.86
SC_2	21,986,238	98.54	1.46	95.02	35.49	89.71
Tumor_1	158,902,922	98.74	1.26	91.04	34.64	86.00
Tumor_2	167,354,016	97.94	2.06	90.75	34.54	80.88

References:

1. Tirosh, I. *et al.* Dissecting the multicellular ecosystem of metastatic melanoma by single-cell RNA-seq. *Science* **352**, 189-196 (2016).
