# Supplementary material for: A novel mouse model demonstrates that oncogenic melanocyte stem cells engender melanoma resembling human disease
Source: Nat Commun. 2019 Nov 4;10:5023. doi: 10.1038/s41467-019-12733-1 (PMC6828673; doi:10.1038/s41467-019-12733-1)
Supplement: Supplementary file 2 — Description of Additional Supplementary Files [file 41467_2019_12733_MOESM2_ESM.docx]

**Description of Supplementary Files**

**File Name:** Supplementary Data 1

**Description:** Top 200 enriched genes in each cluster of the melanoma cells identified in single cell RNA-seq analysis.
