# Supplementary material for: A novel mouse model demonstrates that oncogenic melanocyte stem cells engender melanoma resembling human disease
Source: Nat Commun. 2019 Nov 4;10:5023. doi: 10.1038/s41467-019-12733-1 (PMC6828673; doi:10.1038/s41467-019-12733-1)
Supplement: Supplementary file 4 — Reporting Summary [file 41467_2019_12733_MOESM4_ESM.pdf]

nature research

Corresponding author(s): Mayumi Ito
Last updated by author(s): Sep 9, 2019

Reporting Summary

Nature Research wishes to improve the reproducibility of the work that we publish. This form provides structure for consistency and transparency in reporting. For further information on Nature Research policies, see Authors & Referees and the Editorial Policy Checklist.

Statistics

For all statistical analyses, confirm that the following items are present in the figure legend, table legend, main text, or Methods section.

n/a	Confirmed	
☐	☒	The exact sample size (*n*) for each experimental group/condition, given as a discrete number and unit of measurement
☐	☒	A statement on whether measurements were taken from distinct samples or whether the same sample was measured repeatedly
☐	☒	The statistical test(s) used AND whether they are one- or two-sided<br>*Only common tests should be described solely by name; describe more complex techniques in the Methods section.*
☒	☐	A description of all covariates tested
☒	☐	A description of any assumptions or corrections, such as tests of normality and adjustment for multiple comparisons
☐	☒	A full description of the statistical parameters including central tendency (e.g. means) or other basic estimates (e.g. regression coefficient) AND variation (e.g. standard deviation) or associated estimates of uncertainty (e.g. confidence intervals)
☒	☐	For null hypothesis testing, the test statistic (e.g. *F*, *t*, *r*) with confidence intervals, effect sizes, degrees of freedom and *P* value noted<br>*Give P values as exact values whenever suitable.*
☒	☐	For Bayesian analysis, information on the choice of priors and Markov chain Monte Carlo settings
☒	☐	For hierarchical and complex designs, identification of the appropriate level for tests and full reporting of outcomes
☒	☐	Estimates of effect sizes (e.g. Cohen's *d*, Pearson's *r*), indicating how they were calculated

Our web collection on statistics for biologists contains articles on many of the points above.

Software and code

Policy information about availability of computer code

Data collection	All immunofluorescence were analyzed and photographed with a Nikon Eclipse Ti microscope with NIS-Elements software.<br>Cell sorting was performed on Beckman Coulter MoFlo cell sorter or Sony SY3200 cell sorter.<br>Bulk RNA-seq data were acquired with Illumina Hiseq-2500 sequencer.<br>Single cell RNA-seq were acquired with Illumina HiSeq 4000 as 2×150 paired-end reads.
Data analysis	Immunofluorescence images were analyzed with NIS-Elements software and Adobe Photoshop.<br>Dot and box plots were drawn and t-test were performed using Excel and Prism (version 7).<br>Bulk RNA-seq data were mapped to mouse genome (NCBI37/mm9) by using Bowtie aligner (0.12.9) (Langmead et al., 2009) with v2 and m1 parameters. Mapped reads were assigned to Ensemble gene model (Mus_musculus.NCBIM37.67.gtf) with feature count package (Liao et al., 2014). To perform statistical analysis for significant differential expressed genes, edgeR (3.4.2) (Robinson et al., 2010) was used for the data normalization and comparison between experimental and control groups. The p value was adjusted with FDR method. Significant genes for later analysis was selected as FDR < 0.02 and fold change >4.<br>Single cell RNA-seq data were analyzed using Cell Ranger Single-Cell Software Suite (10x Genomics) to perform sample demultiplexing, barcode processing, and single-cell 3′ gene counting. The cDNA insert was aligned to the mm10/GRCm38 reference genome. Only confidently mapped, non-PCR duplicates with valid barcodes and UMIs were used to generate the gene-barcode matrix. Further analysis and visualization was performed using Seurat, a public available R package containing implementations of commonly used single-cell analytical techniques, including the identification of highly variable genes, dimensionality reduction, standard unsupervised clustering algorithms, and the discovery of differentially expressed genes and markers. For pseudo-temporal analysis, digital gene expression matrices with annotations from Seurat were analyzed by Monocle v2.10.1 (pseudotime analysis). The top 1, 500 genes with highest dispersion (variation/mean) were used to construct the pseudo-temporal trajectory.<br>The GSEA program was downloaded from the BROAD institute website (http://www.broadinstitute.org/gsea/).We compared our pre-ranked gene lists for their enrichment in 4 annotated gene sets acquired from The Molecular Signature Database (MSigDB). change-ranked list of genes.Results of the enrichment analysis were plotted using R software.

For manuscripts utilizing custom algorithms or software that are central to the research but not yet described in published literature, software must be made available to editors/reviewers. We strongly encourage code deposition in a community repository (e.g. GitHub). See the Nature Research guidelines for submitting code & software for further information.

Data

Policy information about availability of data

All manuscripts must include a data availability statement. This statement should provide the following information, where applicable:

- Accession codes, unique identifiers, or web links for publicly available datasets
- A list of figures that have associated raw data
- A description of any restrictions on data availability

The accession number for the single cell RNA-sequencing data reported in this paper Gene Expression Omnibus (NCBI) GEO: GSE96966 (bulk RNA-seq) and GSE113502 (single cell RNA-seq).

Field-specific reporting

Please select the one below that is the best fit for your research. If you are not sure, read the appropriate sections before making your selection.

☒ Life sciences ☐ Behavioural & social sciences ☐ Ecological, evolutionary & environmental sciences

For a reference copy of the document with all sections, see nature.com/documents/nr-reporting-summary-flat.pdf

Life sciences study design

All studies must disclose on these points even when the disclosure is negative.

Sample size	No statistical methods were used to predetermine sample size. All experiments were repeated at least two-three times with similar r esults. For single cell RNA-seq, cells isolated from 3 independent animal repeats were combined before sequencing.
Data exclusions	No animals were excluded from the analysis.
Replication	All experiments were repeated at least three times (3 biological independent experiments/mice) showing similar results, except for bulk RNA-seq analysis, Supplementary Figure 2c and 5c, for which experiments were repeated twice (2 biological independent experiments/mice). For single cell RNA-seq, cells isolated from 3 independent animal repeats were combined before sequencing. All attempts at replication were successful, n is described in legends.
Randomization	The experiments were not randomized because the mice used in this study were selected according to their correct genotype. Sex-specific differences were minimized by including similar number of male and female animals. Age-specific differences were minimized by using mice of comparable age in different experimental groups.
Blinding	The investigators were not blinded to allocation during experiments and outcome assessment. Blinding was not possible as the same investigator processed the animals and analyzed the data.

Reporting for specific materials, systems and methods

We require information from authors about some types of materials, experimental systems and methods used in many studies. Here, indicate whether each material, system or method listed is relevant to your study. If you are not sure if a list item applies to your research, read the appropriate section before selecting a response.

Materials & experimental systems

n/a	Involved in the study	
☐	☒	Antibodies
☒	☐	Eukaryotic cell lines
☒	☐	Palaeontology
☐	☒	Animals and other organisms
☒	☐	Human research participants
☒	☐	Clinical data

Methods

n/a	Involved in the study	
☒	☐	ChIP-seq
☐	☒	Flow cytometry
☒	☐	MRI-based neuroimaging

Antibodies

Antibodies used	Goat polyclonal anti-Dct Santa Cruz Cat#sc-10451<br>Rabbit polyclonal anti-GFP Abcam Cat#ab290<br>Chicken polyclonal anti-GFP Abcam Cat#ab13970<br>Rat monoclonal anti-c-Kit BD Biosciences Cat#553868<br>Goat polyclonal anti-Sox10 Santa Cruz Cat#sc-17342<br>Rabbit polyclonal anti-S100 Dako Cat#Z0311<br>Mouse monoclonal anti-Nestin Abcam Cat#ab6142

	Rabbit polyclonal anti-Tomato Rockland Cat#600-401-379<br>Mouse monoclonal anti-Tomato Thermo Fisher Scientific Cat#MA5-15257<br>Rabbit polyclonal anti-Keratin14 Covance Cat#PRB-155P<br>Rabbit polyclonal anti-Ki67 Abcam Cat#15580<br>Rabbit polyclonal anti pAKT XP Cell Signaling Technology Cat#4060S<br>Mouse monoclonal anti-MITF Abcam Cat#ab12039<br>Mouse monoclonal anti-B-catenin Sigma-Aldrich Cat#C7207<br>Mouse monoclonal anti-E-cadherin BD Biosciences Cat#610181<br>Rabbit polyclonal anti-MCAM Abcam Cat#75769<br>Mouse monoclonal anti-Tubb3 Biolegend Cat#801201<br>Rabbit polyclonal anti-GFAP Dako Cat#Z0334<br>Biotin anti-mouse CD45 Biolegend Cat#103103<br>Rat monoclonal anti-BrdU Abcam Cat#ab6326<br>Donkey anti-goat IgG (H+L) secondary antibody, Alexa Fluor 488 Thermo Fisher Scientific Cat#A-11055<br>Donkey anti-goat IgG (H+L) secondary antibody, Alexa Fluor 594 Thermo Fisher Scientific Cat#A-11058<br>Donkey anti-rabbit IgG (H+L) secondary antibody, Alexa Fluor 488 Thermo Fisher Scientific Cat#A-21206<br>Donkey anti-rabbit IgG (H+L) secondary antibody, Alexa Fluor 594 Thermo Fisher Scientific Cat#A21207<br>Donkey anti-mouse IgG (H+L) secondary antibody, Alexa Fluor 488 Thermo Fisher Scientific Cat#A-21202<br>Donkey anti- mouse IgG (H+L) secondary antibody, Alexa Fluor 594 Thermo Fisher Scientific Cat#A-21203<br>Donkey anti-rat IgG (H+L) highly cross-adsorbed secondary antibody, Alexa Fluor 488 Thermo Fisher Scientific Cat#A-21208<br>Goat anti-chicken IgG (H+L) secondary antibody, Alexa Fluor 488 Thermo Fisher Scientific Cat#A-11039<br>Biotinylated goat anti-rat IgG antibody Vector Laboratories Cat#BA-9400<br>Biotinylated horse anti-mouse IgG antibody Vector Laboratories Cat#BA-2000<br>Streptavidin, Alexa Fluor 488 conjugate Thermo Fisher Scientific Cat#S32354<br>Streptavidin, Alexa Fluor 594 conjugate Thermo Fisher Scientific Cat#S32356<br>Streptavidin, Alexa Fluor 647 conjugate Thermo Fisher Scientific Cat#S32357
Validation	As positive control tissues with known expression of the marker were used. As negative control staining omitting the primary was performed.

Animals and other organisms

Policy information about studies involving animals; ARRIVE guidelines recommended for reporting animal research

Laboratory animals	B6.Cg-Tg(Tyr-cre/ERT2)13Bos/J The Jackson Laboratory Stock#: 012328<br>B6.Cg-Braftm1Mmcm Ptentm1Hwu Tg(Tyr-cre/ERT2)13Bos/BosJ The Jackson Laboratory Stock#: 013590<br>B6;129S6-Gt(ROSA)26Sortm14(CAG-tdTomato)Hze/J The Jackson Laboratory Stock#: 007908<br>Gt(ROSA)26Sortm4(ACTB-tdTomato,-EGFP)Luo/J The Jackson Laboratory Stock#: 007576<br>FVB-Tg(KRT14-rtTA)F42Efu/J The Jackson Laboratory Stock#: 008099<br>C57BL/6-Tg(tetO-EDN1,-lacZ)9Mhus/J The Jackson Laboratory Stock#: 013729<br>J:NU The Jackson Laboratory Stock#: 007850<br>iDCT-GFP NCI Mouse Repository 01XT4<br>c-Kit-CreERT2 (Klein et al., 2013)<br>β-catenin fl(ex3)/+ (Harada et al., 1999)
Wild animals	No wild animals were used in this study.
Field-collected samples	No field-collected samples were used in this study.
Ethics oversight	The Institutional Animal Care and Use Committee (IACUC) at New York University School of Medicine

Note that full information on the approval of the study protocol must also be provided in the manuscript.

Flow Cytometry

Plots

Confirm that:

☒ The axis labels state the marker and fluorochrome used (e.g. CD4-FITC).

☒ The axis scales are clearly visible. Include numbers along axes only for bottom left plot of group (a 'group' is an analysis of identical markers).

☒ All plots are contour plots with outliers or pseudocolor plots.

☒ A numerical value for number of cells or percentage (with statistics) is provided.

Methodology

Sample preparation	Melanocyte stem cells were isolated according to methods described by (Chou et al., 2013; Rabbani et al., 2011). Briefly, mouse back skin was cut into 0.5cmX0.5cm pieces, followed by incubation in 0.25% Trypsin for 2 hr at 37° C. Epidermis was separated from the dermis using forceps and scalpel blades and the epidermis was chopped finely and stirred at RT for 20 min Media A (DMEM, 10% FBS, 1x penicillin/streptomycin) (Morris et al., 2004) to obtain single cell suspension.

	Melanoma cells were isolated by finely mince mouse melanoma tumors with scissors, followed by incubation in Media A with 0.35% Collagenase I for 1 hr, shaken (100 rpm) at 37°C, to obtain single cell suspension.
Instrument	Beckman Coulter MoFlo cell sorter or Sony SY3200 cell sorter
Software	Summit Software was used for collecting data using Beckman Coulter MoFlo cell sorter. Eclipse EC800 Analyzer was used for collecting data using Sony SY3200 cell sorter. Flow cytometry was only used for cell collection in this study.
Cell population abundance	Melanocyte stem cells or melanoma cells derived from them were selected based on the expression of Tomato reporter. Tomato + melanocyte stem cells are usually 0.1-1% of total live single cells in the epithelium compartment, isolated as described above. Tomato+ melanoma cells are usually 50-70% of total live single cells in the tumor, isolated as described above.
Gating strategy	Living cells were selected by forward scatter, side scatter, doublets discrimination and by DAPI dye exclusion. Melanocyte stem cells or melanoma cells derived from them were selected based on the expression of Tomato reporter.

☒ Tick this box to confirm that a figure exemplifying the gating strategy is provided in the Supplementary Information.
